# Microbiota-induced tissue signals regulate ILC3-mediated antigen presentation

**DOI:** 10.1038/s41467-020-15612-2

**Published:** 2020-04-14

**Authors:** Frank Michael Lehmann, Nicole von Burg, Robert Ivanek, Claudia Teufel, Edit Horvath, Annick Peter, Gleb Turchinovich, Daniel Staehli, Tobias Eichlisberger, Mercedes Gomez de Agüero, Mairene Coto-Llerena, Michaela Prchal-Murphy, Veronika Sexl, Mohamed Bentires-Alj, Christoph Mueller, Daniela Finke

**Affiliations:** 1 Department of Biomedicine and University Children’s Hospital of Basel, University of Basel, 4058 Basel, Switzerland; 2 Department of Health Technology, Technical University of Denmark, 2800 Kgs. Lyngby, Denmark; 3 Department of Biomedicine, University of Basel, 4056 Basel, Switzerland; 4 Swiss Institute of Bioinformatics, 4053 Basel, Switzerland; 5 Friedrich Miescher Institute for Biomedical Research, 4058 Basel, Switzerland; 6 Maurice Müller Laboratories, Department for BioMedical Research, Universitätsklinik für Viszerale Chirurgie und Medizin Inselspital, University of Bern, 3010 Bern, Switzerland; 7 Institute of Pharmacology and Toxicology, Department for Biomedical Sciences, University of Veterinary Medicine Vienna, Vienna, Austria; 8 Institute of Pathology, University of Bern, 3008 Bern, Switzerland

**Keywords:** Interleukins, Innate lymphoid cells, T cells, Mucosal immunology

## Abstract

Although group 3 innate lymphoid cells (ILC3s) are efficient inducers of T cell responses in the spleen, they fail to induce CD4^+^ T cell proliferation in the gut. The signals regulating ILC3-T cell responses remain unknown. Here, we show that transcripts associated with MHC II antigen presentation are down-modulated in intestinal natural cytotoxicity receptor (NCR)^−^ ILC3s. Further data implicate microbiota-induced IL-23 as a crucial signal for reversible silencing of MHC II in ILC3s, thereby reducing the capacity of ILC3s to present antigen to T cells in the intestinal mucosa. Moreover, IL-23-mediated MHC II suppression is dependent on mTORC1 and STAT3 phosphorylation in NCR^−^ ILC3s. By contrast, splenic interferon-γ induces MHC II expression and CD4^+^ T cell stimulation by NCR^−^ ILC3s. Our results thus identify biological circuits for tissue-specific regulation of ILC3-dependent T cell responses. These pathways may have implications for inducing or silencing T cell responses in human diseases.

## Abstract (web summary)

Group 3 innate lymphoid cells (ILC3s) promote T cell activation in the spleen but suppress it in the gut. Here, the authors show that this distinct regulation is mediated by gut microbiota-induced IL-23 and IFN-γ, respectively, and, along with the article by Rao et al, this work elucidates how cytokines set context specificity of ILC-T cell crosstalk by regulating ILC antigen presentation.

## Introduction

ILC3s are primarily tissue-resident cells, which rapidly respond to infections and inflammation by cytokine secretion. They express and depend on the transcription factor RAR-related orphan receptor gamma t(RORγt)^1,2^ and can be subdivided into natural cytotoxicity receptor (NCR)^+^ and NCR^−^ cells^3^. Similar to T helper (TH)22 and TH17 cells, ILC3s secrete interleukin (IL)-22 and IL-17^4–6^ and play a role in defense against *Citrobacter rodentium* infection and in tissue regeneration^7–11^. In addition to their function as early cytokine producers, recent analysis has revealed that ILC3 subsets can present antigen (Ag) to CD4^+^ T cells, but the quality and strength of T-cell response is tissue-dependent^12–14^. How ILC3-T-cell responses are regulated remains poorly defined.

In adults, ILC3s are abundant in mucosal tissues, e.g., the small intestine (SI) and colon, and mucosa-associated lymphoid organs^3,15^. In addition, ILC3s are found in the spleen (SP) and peripheral lymph nodes^6,15^. It is now increasingly recognized that ILCs exhibit heterogenous phenotypes across different tissues^16–19^. The exposure to environmental signals including microbial and nutrient-derived metabolites has been suggested to be relevant for the regulation of IL-22 and IL-17 responses of intestinal ILC3s^7,20–23^. The nature of signals that regulate Ag presentation and T-cell stimulation by ILC3s, however, is largely unknown. Moreover, data on a direct comparison of ILC3s among different organs are limited and often based on a sorting strategy not considering subsets. Single-cell transcriptome profiling of SI ILCs revealed that major histocompatibility complex (MHC) class II (MHC II) is mainly found in a NCR^−^ ILC3 subset that lacks the T-box transcription factor T-bet (encoded by *Tbx21*) and is only partially overlapping with IL-22-producing ILC3s^20^. In the SP, Ag-presenting NCR^−^ ILC3s elicit specific CD4^+^ T-cell responses and support the maintenance of CD4^+^ memory T cells^14,24^. In the SI, however, MHC II^+^ ILC3s are considered as negative regulators of commensal bacteria-specific CD4^+^ T-cell responses^12,13,25^. A central question that has not been addressed concerns the tissue-specific signals that regulate the capacity of ILC3 subsets to present Ag to CD4^+^ T cells.

We demonstrate here that NCR^−^ ILC3s isolated from the SP and the SI of mice have a fundamentally distinct transcriptional signature. SP NCR^−^ ILC3s harbor MHC II^+^ ILC3s with the capacity to elicit cognate CD4^+^ T-cell responses. In contrast, the majority of SI NCR^−^ ILC3s lack MHC II and fail to induce T-cell proliferation. This is due to the fact that the gut microbiota and IL-23 inhibit the expression of MHC II on ILC3s via pathways engaging mammalian target of rapamycin complex 1 (mTORC1) and signal transducer and activator of transcription 3 (STAT3). On the contrary, interferon γ (IFN-γ) enhances the expression of MHC II on ILC3s in the SP. Finally, we show that the functional inhibition of ILC3s by the SI environment is reversible both in vitro and in vivo. Together our data uncover the basic molecular requirements of ILC3s to control T-cell responses and highlight the role of the local environment in shaping tissue-specific ILC3-T-cell interactions.

## Results

### SP and SI ILC3s have a different transcriptional signature

**Fig. 1:**
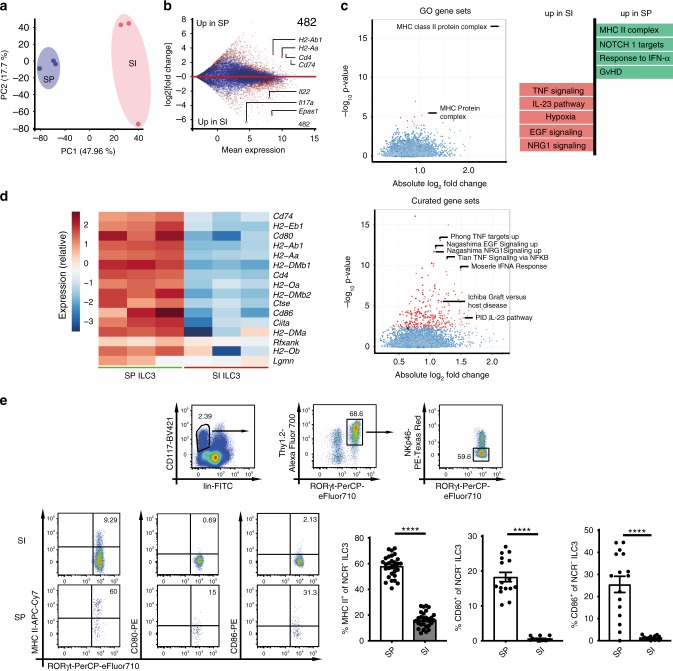
SP and SI NCR^−^ ILC3s exhibit a different transcriptional signature. **a** PCA of RNA sequencing data of SI and SP NCR^*−*^ ILC3s isolated from *Rorγt*^*fm+*^ mice. Cells were sort-purified as depicted in Supplementary Fig. 1a. **b** Mean expression and log 2(fold change) of all detected genes. Genes with a significant difference are highlighted in red (FDR < 0.05). Numbers indicate the total amount of genes significantly higher expressed (log2(fold change)>1.5) in SP ILC3s or SI ILC3s. **c** Gene set enrichment analysis of gene ontology (GO) and curated gene sets. Gene sets with a significant difference are highlighted in red (FDR < 0.05). **d** Heatmap of genes associated with MHC II Ag presentation. **e** CD117^+^lin^*−*^Thy1.2^+^RORγt^+^NKp46^*−*^ ILC3s were analyzed for surface expression of MHC II (*n* = 29(SP) and *n* = 27(SI) mice), CD80 (*n* = 16(SP) and *n* = 14(SI) mice), and CD86 (*n* = 16(SP) and *n* = 14(SI) mice). Six to eight independent experiments. Each symbol represents a sample and the bar graph represents the mean ± s.e.m. ****P ≤ 0.0001, calculated with unpaired two-tailed Student’s *t* test. Source data are provided as a Source Data File.

SP and SI ILC3s differ in their capacity to induce Ag-dependent CD4^+^ T-cell responses^12,14^. To study the tissue-specific regulation of SP vs. SI NCR^−^ ILC3 transcriptional programs, we profiled the transcriptome of the two subsets. To this end, lin^−^CD117^+^Thy1.2^+^eYFP^+^ cells from SP and SI of *RORc(γt)-Cre*^*tg*^ *Rosa26R*^*eYFP/+*^ (*Rorγt*^*fm+*^) mice were sort-purified and subjected to RNA-sequencing (Supplementary Fig. 1a). The principal component analysis (PCA) of SP and SI NCR^−^ ILC3s revealed a different transcriptional profile of the two subsets (Fig. 1a). A total set of 1286 genes was differentially expressed with specific signature of SP and SI ILC3s for genes encoding transcription factors (e.g., *Epas1*, *Arnt2*, or *Runx1t1*), cytokines (e.g., *Il22* and *Il17a*) and genes involved in IL-23, hypoxia, or IFN-α responses (Fig. 1b, c, Supplementary Fig. 1b, c). Importantly, transcripts relevant for MHC II-dependent Ag presentation and costimulation (e.g., *H2-Aa*, *H2-Ab*, *Cd74*, *Cd80*, *Cd86,* and *Ctse*) were enriched in SP ILC3s (Fig. 1d). Flow cytometry analysis confirmed the enhanced expression of MHC II, CD80, and CD86 of SP NCR^−^ ILC3s (Fig. 1e). A subset of naive MHC II^+^ and MHC II^−^NCR^−^ ILC3s produced IL-22 (Supplementary Fig. 1d). In line with previous publications only the C-C chemokine receptor type 6 (CCR6)^+^ subset of NCR^−^ ILC3s expressed MHC II (Supplementary Fig. 1d)^12,20^.

### SP and SI ILC3s differ in their capacity to activate T cells

As transcripts required for Ag presentation were enriched in SP ILC3s, we measured the capacity of activated SP and SI ILC3s to process and present Ag and to induce CD4^+^ T-cell activation and proliferation. SP and SI NCR^−^ ILC3s from *Rag2*^*−/−*^ mice (Supplementary Fig. 2a, b) and bone marrow-derived dendritic cells (BMDCs) as positive control were stimulated with IL-1β and cultured in the presence of Ovalbumin (Ova) protein or peptide with Ova-specific T-cell receptor (TCR) transgenic CD4^+^ T cells (*OT-II*^*tg*^ CD4^+^ T cells). Pre-activation of Ag-presenting cells (APCs) was chosen to simulate immunogenic conditions under which T-cell responses toward foreign Ag are elicited in vivo. IL-1β boosts the capacity of SP ILC3s to induce T-cell responses in vitro by upregulation of CD80, CD86 and MHC II^14^. IL-1β also induced the expression of *Tnfsf4* and its product OX40L by SP and SI ILC3s (Supplementary Fig. 2c, d).

**Fig. 2:**
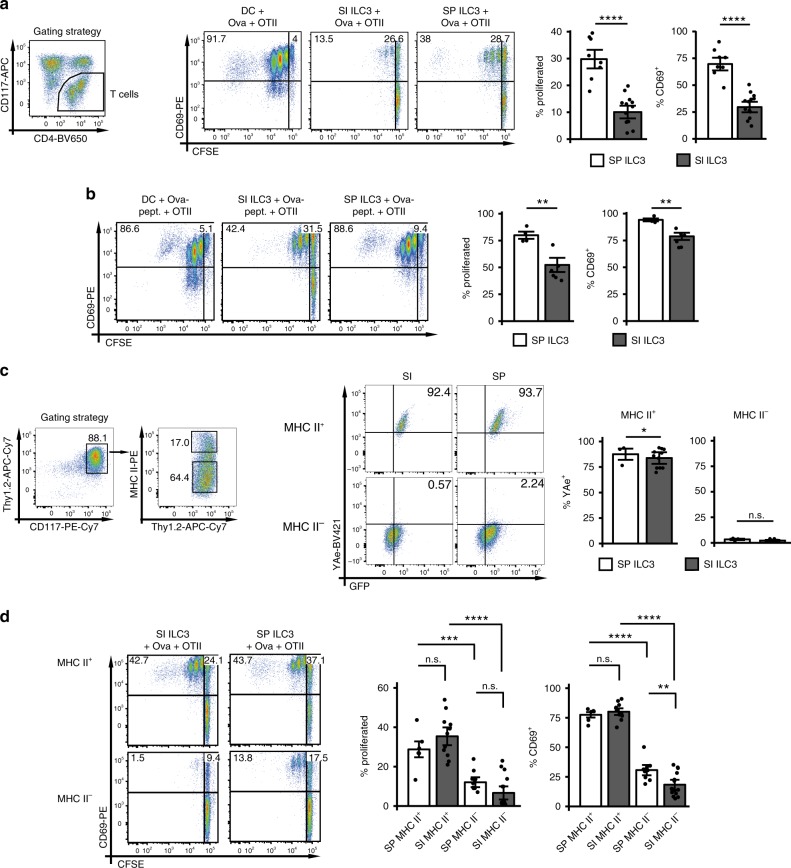
SP and SI NCR^−^ ILC3s differ in their capacity to stimulate CD4^+^ T cells. Naive CFSE-labeled *OT-II*^*tg*^ CD4^+^ T cells were cultured either with 5 × 10^4^ BMDCs, SP or SI ILC3s (*Rag2*^*−/−*^) pre-activated with IL-1β, in the presence of Ova protein (**a**) or peptide (**b**) for 72 h. ILC3s were sorted as depicted in Supplementary Fig. 2a. T cells were gated as CD3^+^ or CD117^−^. In (**a**), *n* = 8 (SP) and *n* = 13 (SI) distinct samples. Six independent experiments. In (**b**), *n* = 4 (SP) and *n* = 6 (SI) distinct samples. Three independent experiments. **c** SP or SI ILC3s (*Rag2*^*−/−*^) were cultured with Eα-GFP protein for 72 h. ILC3s were sorted as depicted in Supplementary Fig. 2a. Surface presentation of Eα peptide was analyzed by flow cytometry with antibody clone YAe. ILC3s were gated either on MHC II^+^ or MHC II^−^CD117^+^Thy1.2^+^ cells (gating strategy is depicted for SI ILC3s). *n* = 3 (SP) and *n* = 11 (SI) distinct samples of three independent experiments. **d** MHC II^+^ (*n* = 6 (SP) and *n* = 11 (SI) distinct samples) and MHC II^−^ (*n* = 10 (SP) and *n* = 15 (SI) distinct samples) ILC3s from SP and SI (*Rag2*^*−/−*^) were stimulated with IL-1β and used for T-cell stimulation in the presence of Ova protein. Five independent experiments. ILC3s were sorted as depicted in Supplementary Fig. 2a, e. T cells were gated as CD3^+^ or CD117^−^. Each symbol represents a sample and the bar graph represents the mean ± s.e.m. n.s., not significant; *P ≤ 0.05; **P ≤ 0.01; ***P ≤ 0.001; ****P ≤ 0.0001, calculated with mixed-effects models (two-sided) using lmerTest. Source data are provided as a Source Data File.

In the presence of either Ova protein or peptide SP NCR^−^ ILC3s induced significant CD69 upregulation and proliferation of *OT-II*^*tg*^ CD4^+^ T cells (Fig. 2a, b). Only a weak T-cell proliferation was observed with SI ILC3s and Ova protein, whereas almost 50% of T cells proliferated with Ova peptide. The observed difference between SP and SI NCR^−^ ILC3s might be explained by two potential mechanisms: (I) SI NCR^−^ ILC3s are less efficient at Ag uptake and processing and/or (II) NCR^−^ ILC3s with properties of APCs are enriched in the SP. The finding that a higher percentage of freshly isolated SP NCR^−^ ILC3s expressed MHC II, CD80, and CD86 as compared with SI NCR^−^ ILC3s (Fig. 1e) supports the latter hypothesis. To explore this further, we studied the protein processing capacity of MHC II^+^ and MHC II^−^ NCR^−^ ILC3s from the SI and the SP (Fig. 2c). For this purpose, ILC3s from *Rag2*^*−/−*^ mice were cultured with Eα-GFP protein. Eα-peptide presented by MHC II molecules was detected by staining with the antibody clone YAe. As expected the YAe antibody did not stain MHC II^−^ ILC3s. Interestingly, comparable frequencies of SI and SP MHC II^+^ NCR^−^ ILC3s presented the Eα peptide. Sort-purified SP and SI MHC II^+^ ILC3s were also equally efficient at inducing T-cell activation and proliferation in the presence of Ova protein (Fig. 2d and Supplementary Fig. 2e). MHC II^−^ SP ILC3s were superior of MHC II^−^ SI ILC3s at inducing T-cell activation, most likely because IL-1β stimulation induced MHC II expression in MHC II^−^-sorted SP ILC3s, but not in SI ILC3s (Supplementary Fig. 2f)^14^. Together these data demonstrate that both SP and SI harbor a MHC II^+^ ILC3 subset that equally processes and presents Ag and induces proliferation of CD4^+^ T cells. Furthermore, this subset is significantly enriched in SP NCR^−^ ILC3s ex vivo and after in vitro stimulation with IL-1β.

Since Ova protein pulsed DCs exceeded ILC3s in their capacity to induce T-cell proliferation (Fig. 2a, b), we asked whether DCs and ILC3s also differ in the quality of T-cell responses. Therefore, the cytokine production of *OT-II*^*tg*^ CD4^+^ T cells in co-cultures with pre-activated DCs, SP ILC3s and SI ILC3s was measured. T cells stimulated with ILC3s produced less IFN-γ and more TNF (Supplementary Fig. 2g) as compared with co-cultures with Ag-pulsed DC. The IL-22 secretion of T cells was comparable when ILC3s or DCs were added as APCs (Supplementary Fig. 2g).

It has been previously reported that naive SI ILC3s induce T-cell death of commensal bacteria-specific T cells at steady state^13^. We therefore performed a SI ILC3-CD4^+^ T cell assay and incubated T cells with Annexin V which stains apoptotic cells. The percentage of Annexin V^+^ *OT-II*^*tg*^ CD4^+^ T cells after co-culture with IL-1β-pre-activated SI ILC3s and Ova Ag was reduced as compared with controls without Ova (Supplementary Fig. 2h). It is hence possible that the activation state and the type of Ag determine the effect of ILC3s on T-cell death.

### Tissue localization determines the APC phenotype of ILC3s

**Fig. 3:**
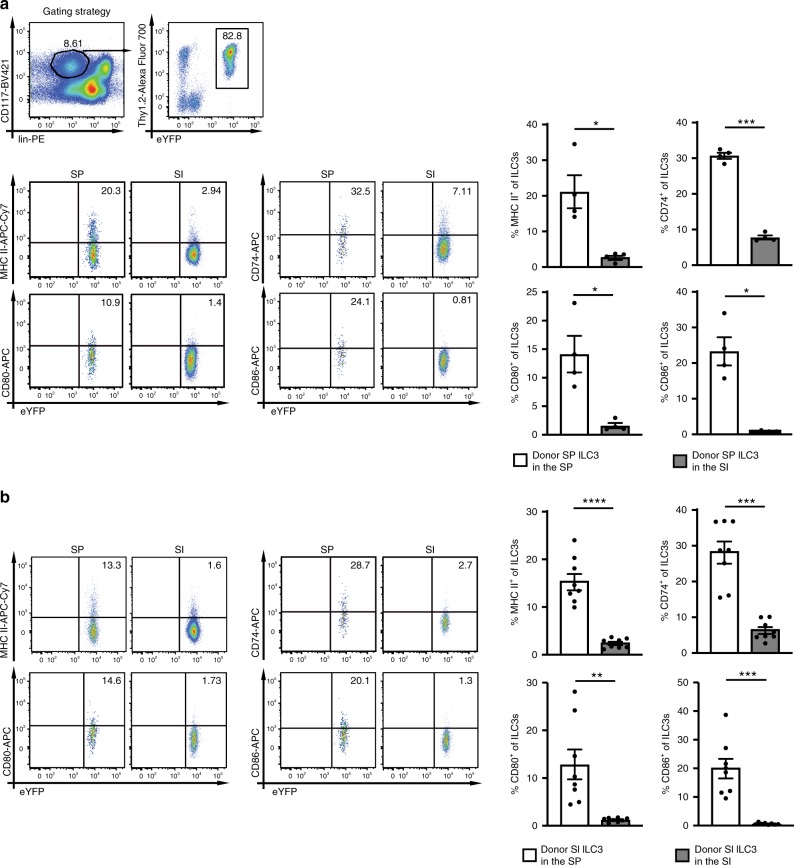
The tissue localization determines the frequency of ILC3s with an APC phenotype. SP ILC3s (**a**) or SI ILC3s (**b**) from *Rorγt*^*fm+*^*Rag2*^*−/−*^ mice were i.v. injected into *Rag2*^*−/−*^*Il2rg*^*−/−*^ mice. Cells were sort-purified as depicted in Supplementary Fig. 1a. Five weeks after transfer the expression of MHC II, CD80, CD86, and CD74 on donor derived ILC3s in the SP and SI of recipient mice was analyzed. ILC3s were gated as depicted in (**a**). **a** *n* = 4 distinct samples of four independent experiments and **b** *n* = 8 distinct samples of five independent experiments. Each symbol represents a sample and the bar graph represents the mean ± s.e.m. *P ≤ 0.05; **P ≤ 0.01; ***P ≤ 0.001; ****P ≤ 0.0001, calculated with two-tailed paired Student’s *t* test. Source data are provided as a Source Data File.

To analyse whether the APC properties of ILC3s are fixed or can be altered by environmental signals, SP and SI ILC3s from *Rorγt*^*fm+*^*Rag2*^*−/−*^ mice were adoptively transferred into *Rag2*^*−/−*^*Il2rg*^*−/−*^ mice. Five weeks after transfer, eYFP^+^ donor ILC3s isolated from the SP and the SI of recipient mice were analyzed for the expression of CD80, CD86, CD74, and MHC II. There was no tissue-origin-specific bias of ILC3s homing to SP and SI. Importantly, SP donor ILC3s isolated from the SI of recipients had down-modulated CD80, CD86, CD74, and MHC II (Fig. 3a). Conversely, SI donor ILC3s isolated from the SP of recipients had upregulated these molecules (Fig. 3b). Similar results were obtained 7 days after adoptive transfer of total splenocytes or intestinal lymphocytes into *Rag2*^*−/−*^*Il2rg*^*−/−*^ mice (Supplementary Fig. 3a, b). In addition, the retention marker CD69 was downregulated in SI ILC3s after migration to the SP and upregulated in SP ILC3s after migration to the SI.

In order to test if retention or survival of MHC II^+^ ILC3s was superior in the SP, SI CD45.1^+^MHC II^−^ and SI eYFP^+^MHC II^+^ ILC3s were injected into *Rag2*^*−/−*^*Il2rg*^*−/−*^ mice (Supplementary Fig. 3c, d). Five weeks later the two donor cell subsets were discriminated by anti-CD45.1 staining and eYFP. The input ratios of donor ILC3 subsets did not differ from the ratios in recipient SP. This strongly suggests that MHC II^+^ and MHC II^−^ ILC3s were comparable in retention or survival in the SP (Supplementary Fig. 3d). In line with previous results MHC II^+^ ILC3s lost MHC II expression in the intestine and MHC II^−^ ILC3s gained MHC II expression in the SP (Supplementary Fig. 3e).

Altogether the SI environment suppressed the expression of molecules required for MHC II Ag presentation and T-cell activation. Our data suggest that NCR^−^ ILC3s exhibit certain plasticity and that tissue-specific factors condition the capacity of ILC3s to act as APCs for CD4^+^ T cells.

### In vitro culture reverses functional polarization of ILC3s

**Fig. 4:**
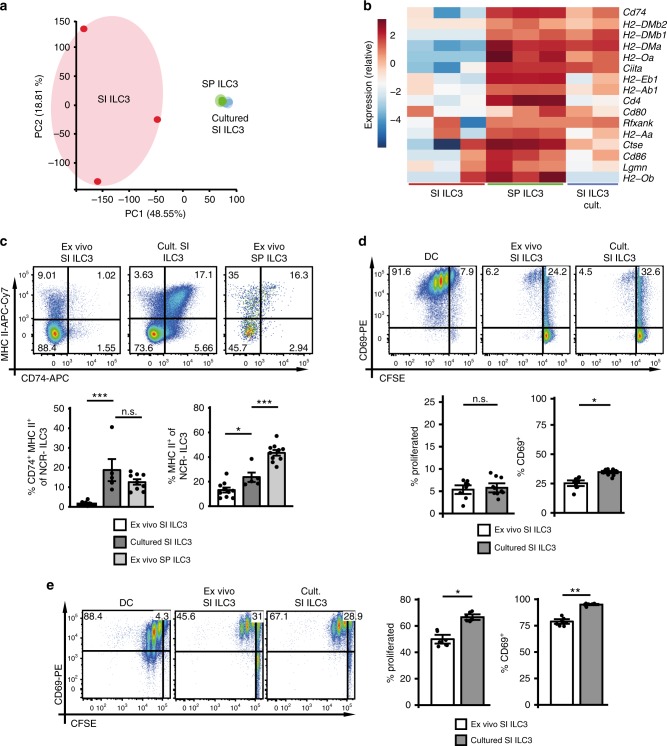
MHC II downregulation of SI ILC3s is reversible upon in vitro culture. CD117^+^Thy1.2^+^lin^−^ (CD3ε, CD8α, CD11b, CD11c, CD19, B220, Gr-1, TCR-β, TCR-γ/δ, TER119, NK1.1) KLRG1^−^ ILC3s were isolated from SP or SI of *Rag2*^*−/−*^ mice (Supplementary Fig. 2a). Freshly isolated (ex vivo) SP (green) or SI ILC3s (red) and SI ILC3s cultured for 7 days with IL-2, IL-7, and SCF (blue) were used for RNA sequencing. **a** PCA of RNA sequencing samples. **b** Heatmap of genes associated with MHC II Ag presentation. **c** Expression of MHC II and CD74 by cultured ILC3s (*n* = 5 distinct samples) or ex vivo SI (*n* = 9–10 distinct samples) and SP (*n* = 9–10 distinct samples) ILC3s of *Rag2*^*−/−*^ mice. Five independent experiments. **d**, **e** Naive CFSE-labeled *OT-II*^*tg*^ CD4^+^ T cells were cultured either with 5 × 10^4^ BMDCs, ex vivo or cultured SI ILC3s from *Rag2*^*−/−*^ mice in the presence of Ova protein (**d**) or peptide (**e**). In (**d**), *n* = 9 (ex vivo SI ILC3) and *n* = 10 (cultured SI ILC3) distinct samples of four independent experiments. In (**e**), *n* = 8 distinct samples of three independent experiments. T cells were gated as in Fig. 2. Each symbol represents a sample and the bar graph represents the mean ± .e.m. n.s., not significant; *P ≤ 0.05; **P ≤ 0.01; ***P ≤ 0.001, calculated with one-way ANOVA (two-tailed) and Bonferroni’s multiple comparisons test (**c**) or with mixed-effects models (two-sided) using lmerTest (**d** and **e**). Source data are provided as a Source Data File.

Our in vivo data showed that SI NCR^−^ ILC3s, when migrating to the SP, upregulated molecules required for Ag presentation and activation of CD4^+^ T cells. To further explore their plasticity, sort-purified SI ILC3s from *Rag2*^*−/−*^ mice were cultured with IL-2, IL-7, and stem cell factor (SCF) for 7 days and used for RNA sequencing, flow cytometric analysis and stimulation of *OT-II*^*tg*^ CD4^+^ T cells. PCA of RNA sequencing data showed that samples from cultured SI ILC3s clustered with freshly isolated (ex vivo) SP ILC3 samples (Fig. 4a). Importantly, transcripts associated with MHC II Ag presentation were upregulated in SI ILC3s after culture (Fig. 4b). In addition, in vitro culture of SI ILC3s resulted in increased MHC II protein expression and accumulation of a CD74^+^ MHC II^+^ population of NCR^−^ ILC3s, a subset existing only at low frequency within ex vivo SI ILC3s (Fig. 4c). These data demonstrate that SI ILC3s acquire a SP-like phenotype upon in vitro culture.

To test the capacity of cultured SI NCR^−^ ILC3s to present Ag and stimulate CD4^+^ T cells, ex vivo and cultured SI ILC3s were incubated with *OT-II*^*tg*^ CD4^+^ T cells and Ova protein (Fig. 4d) or Ova peptide (Fig. 4e). Our data show that cultured SI ILC3s were significantly better at CD4^+^ T-cell stimulation compared with ex vivo SI ILC3s, suggesting that environmental factors in the intestine may negatively affect the Ag presentation capacity of ILC3s.

### IL-23 negatively regulates Ag presentation by ILC3s

**Fig. 5:**
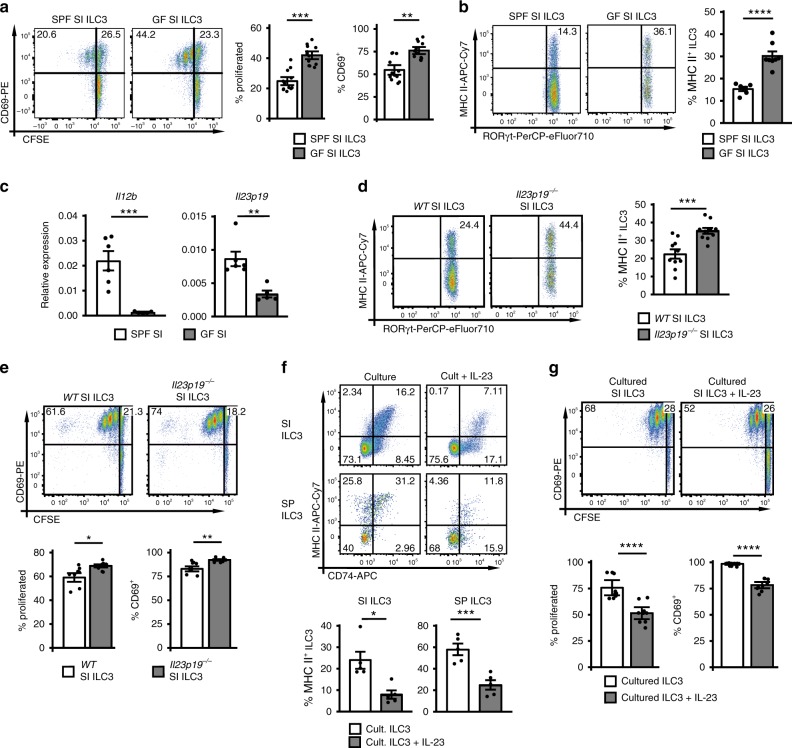
The microbiota and IL-23 negatively regulate Ag presentation by ILC3s. **a** CFSE-labeled *OT-II*^*tg*^ CD4^+^ T cells were stimulated with 1 × 10^4^ SPF or GF ILC3s (*Rag*^*−/−*^ background) in the presence of Ova peptide. *n* = 12 distinct samples of three independent experiments. ILC3s were sorted as depicted in Supplementary Fig. 2a. **b** MHC II expression of SI ILC3s from SPF (*n* = 6 mice) and GF (*n* = 8 mice) mice on *Rag*^*−/−*^ background. NCR^−^ ILC3s were gated as shown in Fig. 1. Two independent experiments. **c** Relative expression of *Il12b* and *Il23p19* in the terminal ileum of SPF (*n* = 6 mice) and GF (*n* = 5 mice) mice on *Rag*^*−/−*^ background analyzed by qRT PCR. Two independent experiments. **d** MHC II expression of SI ILC3s from WT and *Il23p19*^*−/−*^ mice (*n* = 10 mice). NCR^−^ ILC3s were gated as shown in Fig. 1. Five independent experiments. **e** Naive CFSE-labeled *OT-II*^*tg*^ CD4^+^ T cells were cultured either with 5 × 10^4^ SI ILC3s of WT (*n* = 8 distinct samples) or *Il23p19*^*−/−*^ (*n* = 10 distinct samples) mice in the presence of Ova peptide. Four independent experiments. ILC3s were sorted as depicted in Supplementary Fig. 2a. **f** Surface expression of MHC II and CD74 on ILC3s of *Rag2*^*−/−*^ mice cultured 7 days with or without IL-23 in addition to IL-2, IL-7, and SCF (*n* = 5 distinct samples). NCR^−^ ILC3s were gated as shown in Fig. 1. Five independent experiments. **g** SI ILC3s were sort-purified from *Rag2*^*−/−*^ mice (Supplementary Fig. 2a) and cultured 7 days with or without IL-23 in addition to IL-2, IL-7, and SCF. CFSE-labeled *OT-II*^*tg*^ CD4^+^ T cells were stimulated with 5 × 10^4^ cultured ILC3s in the presence of Ova peptide. *n* = 9 distinct samples of three independent experiments. Each symbol represents a sample and the bar graph represents the mean ± s.e.m. *P ≤ 0.05; **P ≤ 0.01; ***P ≤ 0.001; ****P ≤ 0.0001, calculated with mixed-effects models (two-sided) using lmerTest (**a**, **e**, and **g**) or two-tailed unpaired (**b**, **c** *Il12b*, and **d**) or two-tailed paired (**f**) Student’s *t* test or two-tailed Mann–Whitney test (**c** *Il23p19*). Source data are provided as a Source Data File.

To investigate whether microbial products negatively regulated the capacity of SI ILC3s to induce T-cell responses, ILC3s from germ-free (GF) mice were assessed for their ability to stimulate CD4^+^ T cells in vitro and to express MHC II. SI NCR^−^ ILC3s from GF mice were significantly better at inducing Ova-specific CD4^+^ T-cell proliferation in vitro and had a higher expression of MHC II as compared with NCR^−^ ILC3s from conventional specific pathogen-free (SPF) mice (Fig. 5a, b and Supplementary Fig. 8). Together, in the absence of microbiota the capacity of SI ILC3s to stimulate CD4^+^ T cells was enhanced.

Interestingly, gnotobiotic sDMDMm2 mice colonized with a mixture of 12 bacterial strains covering the five major phyla of procaryotes (see Table 1) showed no increased expression of MHC II on SI ILC3s as compared with conventional SPF mice, indicating that these bacteria were sufficient to repress MHC II expression (Supplementary Figs. 4a and 8)^26^.

**Table 1:** Microbial organisms used for sDMDMm2 mice.

Organism	DSM no.
*Lachnoclostridium* sp. YL32	DSM 26114
*Ruminiclostridium* sp. KB18	DSM 26090
*Bacteroides* sp. I48	DSM 26085
*Parabacteroides* sp. YL27	DSM 28989
*Burkholderiales* bacterium YL45	DSM 26109
*Erysipelotrichaceae* bacterium I46	DSM 26113
*Blautia* sp. YL58	DSM 26115
*Flavonifractor plautii* YL31	DSM 26117
*Bifidobacterium animalis* subsp. *animalis* YL2	DSM 26074
*Lactobacillus reuteri* I49	DSM 32035
*Akkermansia muciniphila* YL44	DSM 26127
*Enterococcus faecalis* KB1	DSM 32036

ILC3s isolated from *RORc(γt)-Cre*^*tg*^*Myd88*^*fl/fl*^*(Myd88*^*ILC3−/−*^*)* mice and littermate controls expressed similar MHC II levels indicating that TLR signaling via MyD88 was not responsible for downregulation of MHC II (Supplementary Figs. 4b and 8).

Our observations prompted us to study the effect of IL-23 on MHC II expression of ILC3s, since gut microbes can induce the expression of IL-23 by dendritic cells (DCs)^27^. Indeed, *Il23p19* expression was significantly higher in the SI compared with the SP of SPF mice (Supplementary Fig. 5a). In line with a previous report, the expression of *II12b* and *Il23p19* encoding the subunits of IL-23 were reduced in the terminal ileum of GF compared with SPF mice (Fig. 5c)^28^. To analyse whether IL-23 is involved in shaping the MHC II^+^ to MHC II^−^ ratio of NCR^−^ ILC3s in the SI, IL-23 deficient (*Il23p19*^*−/−*^) mice were used. Interestingly, a higher percentage of MHC II^+^ cells was found in SI NCR^−^ ILC3s of *Il23p19*^*−/−*^ compared with wild-type (WT) mice (Fig. 5d and Supplementary Fig. 8). IL-23 deficiency did not increase the expression of MHC II on ILC3s in the spleen (Supplementary Fig. 5b and 8) most likely because IL-23 is not abundant in the spleen. CD80 and CD86 expression was not significantly affected by the lack of IL-23 (Supplementary Fig. 5c). SI ILC3s from *Il23p19*^*−/−*^ mice showed a significant increase in their capacity to induce Ova-specific *OT-II*^*tg*^ CD4^+^ T-cell responses (Fig. 5e and Supplementary Fig. 5d). Conversely MHC II expression was reduced in WT SI or SP ILC3s after in vitro culture with IL-23 for 7 days (Fig. 5f). IL-23 also repressed the MHC II expression of SI ILC3s isolated from *Il23p19*^*−/−*^ mice, indicating that ILC3s have no intrinsic defect in *Il23p19*^*−/−*^ mice (Supplementary Fig. 5e). Finally, IL-23 added to 7 day cultures of SI ILC3s diminished their capacity to stimulate *OT-II*^*tg*^ CD4^+^ T cells (Fig. 5g, Supplementary Fig. 5f, g).

Collectively these data suggest that microbe-induced IL-23 negatively affects the capacity of NCR^−^ ILC3s to induce CD4^+^ T-cell responses.

### IL-23 reduces MHC II expression through mTORC1 and STAT3

**Fig. 6:**
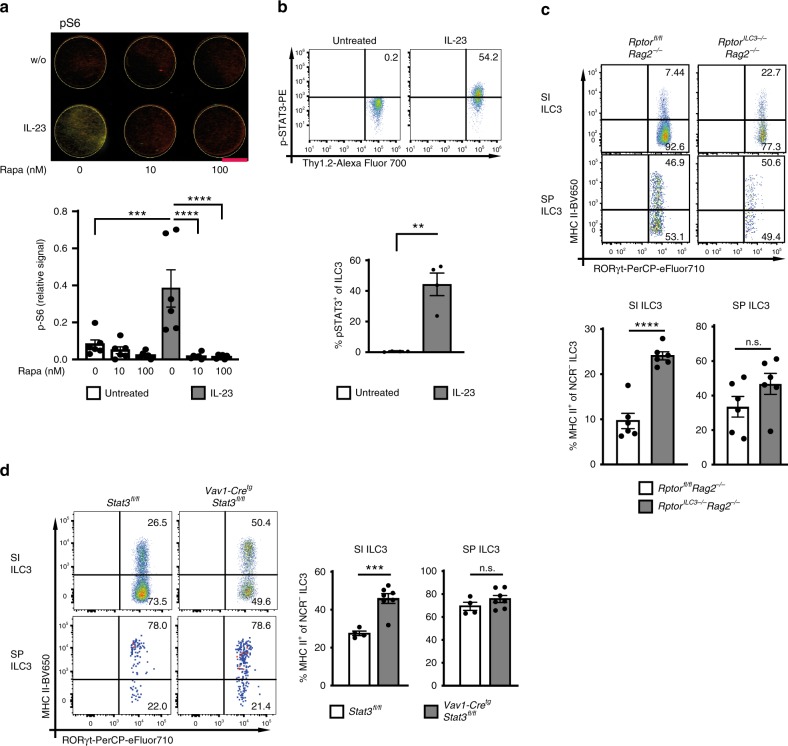
IL-23 reduces MHC II expression through activation of mTORC1 and STAT3. **a** Phosphorylation of S6 of SI ILC3s (sorted from *Flt3L*^*tg*^ mice) after 48 h stimulation with or without IL-23 and 0, 10, or 100 nM Rapamycin (red = tubulin and green = p-S6). ILC3s were sorted as depicted in Supplementary Fig. 2a. Indicated is the relative phosphorylation of S6 (fluorescence signal for p-S6 normalized by fluorescence signal for tubulin). The indicated bar represents 3 mm. *n* = 6 distinct samples of five independent experiments. **b** Phosphorylation of STAT3(Tyr705) of sort-purified SI ILC3s (Supplementary Fig. 2a) from *Rag2*^*−/−*^ mice after 20 min stimulation with or without IL-23. *n* = 4 distinct samples of four independent experiments. **c** MHC II expression of SI and SP ILC3s from *Rptor*^*fl/fl*^*Rag2*^*−/−*^ and *Rptor*^*ILC3−/−*^*Rag2*^*−/−*^ mice. NCR^−^ ILC3s were gated as shown in Fig. 1. *n* = 6 mice of two independent experiments. **d** MHC II expression of SI and SP ILC3s from *Stat3*^*fl/fl*^ (*n* = 4 mice) and *Vav1-Cre*^tg^*Stat3*^*fl/fl*^ (*n* = 7 mice) mice. NCR^−^ ILC3s were gated as shown in Fig. 1. Two independent experiments. Each symbol represents a sample and the bar graph represents the mean ± s.e.m. n.s. not significant; **P ≤ 0.01; ***P ≤ 0.001; ****P ≤ 0.0001, calculated with one-way ANOVA (two-tailed) and Bonferroni’s multiple comparisons test (**a**) or two-tailed paired (**b**) or two-tailed unpaired (**c** and **d**) Student’s *t* test. Source data are provided as a Source Data File.

mTORC1 and STAT3 have been shown to be activated in innate immune cells upon IL-23 stimulation^29–31^. We therefore asked, whether IL-23-mediated silencing of MHC II in SI ILC3s requires mTORC1 and STAT3 signaling. IL-23 stimulation of NCR^−^ ILC3s induced phosphorylation of the mTORC1 signaling kinase S6, mTOR and of STAT3 (Fig. 6a, b and Supplementary Fig. 6a). When ILC3s were incubated with Rapamycin or isolated from mice with a conditional deletion of *Rptor* in ILC3s (*Rptor*^*ILC3−/−*^, *Rag2*^*−/−*^ background) (Fig. 6a and Supplementary Fig. 6a), IL-23 failed to activate mTORC1. Interestingly, the percentage of ex vivo MHC II^+^ SI NCR^−^ ILC3s from *Rptor*^*ILC3−/−*^ mice was twice as high as in control mice (*Rptor*^*fl/fl*^) suggesting that IL-23-mediated mTORC1 signaling suppressed the expression of MHC II on SI ILC3s (Fig. 6c and Supplementary Fig. 8). This was confirmed by stimulating SI and SP ILC3 from *Rptor*^*ILC3−/−*^ mice for 7 days with IL-23 in vitro (Supplementary Fig. 6b). In the SI of mice with a *Stat3* deletion in hematopoietic cells (*VAV1-Cre*^*tg*^*Stat3*^*fl/fl*^) a significantly higher percentage of MHC II^+^ NCR^−^ ILC3s was observed as compared with control mice (Fig. 6d and Supplementary Fig. 8). Notably, in vivo deletion of *Il23p19*, *Rptor* or *Stat3* had no effect on the MHC II^+^ NCR^−^ ILC3s percentage in the SP further emphasizing the fact that MHC II downregulation was specific for the gut (Supplementary Fig. 8).

It was recently shown that IL-23 promotes dermal γδ T-cell differentiation and effector functions via STAT3 phosphorylation independently of the mTOR pathway^32^. Strikingly, IL-23-activated ILC3s from *Rptor*^*ILC3−/−*^ mice showed a reduced STAT3 phosphorylation indicating that in ILC3s the IL-23/STAT3 axis was partially dependent on mTORC1 (Supplementary Fig. 6c). Following IL-23-stimulation S6 phosphorylation was almost normal in *Stat3*-deficient ILC3s as compared with controls indicating that the majority of IL-23-induced mTORC1 signaling occurs independently of STAT3 (Supplementary Fig. 6d). Together these data demonstrate that the IL-23-mTORC1-STAT3 axis mediates downregulation of MHC II in SI ILC3s.

### IFN-γ positively regulates MHC II expression of ILC3s

**Fig. 7:**
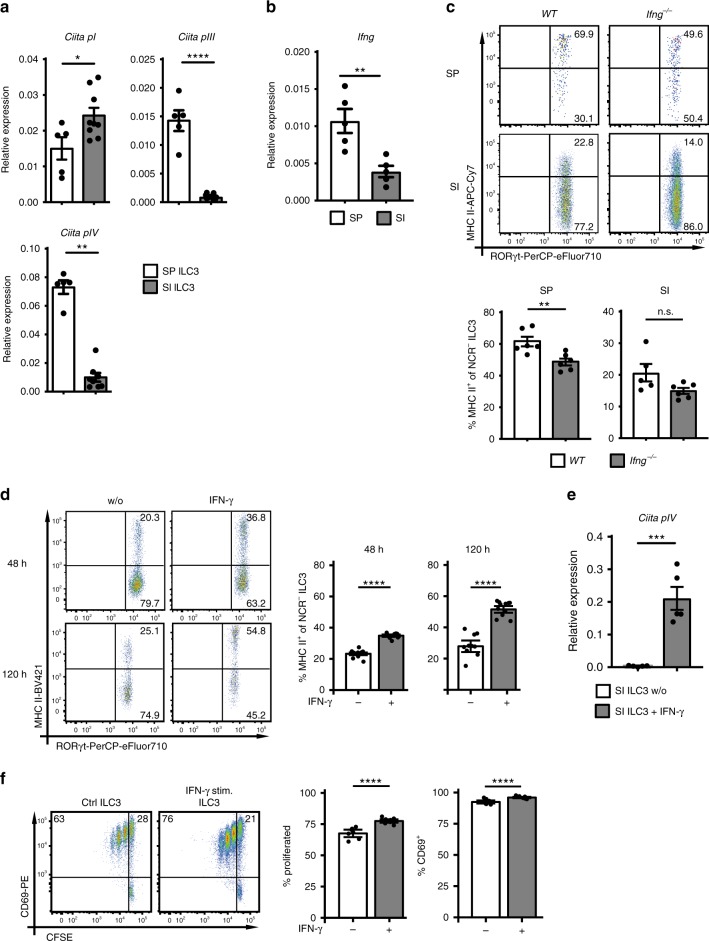
IFN-γ positively regulates MHC II expression of ILC3s. **a** Normalized expression of *Ciita* mRNA isoforms regulated by promoters *pI* (*n* = 5 distinct SP ILC3 samples and *n* = 9 distinct SI ILC3 samples)*, pIII* (*n* = 5 distinct SP ILC3 samples and *n* = 9 distinct SI ILC3 samples), and *pIV* (*n* = 5 distinct SP ILC3 samples and *n* = 8 distinct SI ILC3 samples) in SP and SI ILC3s. ILC3s were sorted as depicted in Supplementary Fig. 2a. Five independent experiments. **b** Expression of IFN-γ in the SP and the SI of WT mice. *n* = 5 mice of two independent experiments. **c** MHC II expression of SI ILC3s from WT (*n* = 6 distinct samples) and *Ifng*^*−/−*^ (*n* = 6 distinct samples) mice. NCR^−^ ILC3s were gated as shown in Fig. 1. Two independent experiments. **d** MHC II expression of SI ILC3s (*Rag2*^*−/−*^) stimulated for 48 h (*n* = 9 distinct samples of three independent experiments) or 120 h (*n* = 10 (w/o) distinct samples and *n* = 11 (IFN-γ stimulated) distinct samples of four independent experiments) with or without (w/o) IFN-γ. ILC3s were sorted as depicted in Supplementary Fig. 2a. **e** Normalized expression of *Ciita* mRNA isoform regulated by promoter *pIV* in SI ILC3s stimulated with or w/o IFN-γ for 120 h. *n* = 5 distinct samples of five independent experiments. ILC3s were sorted as depicted in Supplementary Fig. 2a. **f** SI ILC3s (sorted from *Rag2*^*−/−*^ as depicted in Supplementary Fig. 2a) were stimulated for 120 h with or w/o IFN-γ. Naive CFSE-labeled *OT-II*^*tg*^ CD4^+^ T cells were cultured with 1 × 10^4^ IFN-γ stimulated or unstimulated ILC3s in the presence of Ova peptide. *n* = 7 distinct samples of three independent experiments. Each symbol represents a sample and the bar graph represents the mean ± s.e.m. n.s., not significant; *P ≤ 0.05; **P ≤ 0.01; ***P ≤ 0.001; ****P ≤ 0.0001, calculated with two-tailed unpaired Student’s *t* test (**a** *Ciita pI* and *pIII*, **b**, **c**, and **e**), Mann–Whitney test (**a** *Ciita pIV*) or with mixed-effects models (two-sided) using lmerTest (**d** and **f**). Source data are provided as a Source Data File.

Adoptively transferred SI ILC3s displayed increased levels of MHC II in the SP of recipient mice (Fig. 3b). Since the major transcriptional regulator of MHC II expression is CIITA (class II MHC transactivator), these results suggest that splenic factors may induce or intestinal factors may repress CIITA. We therefore analyzed the expression of the three mRNA isoforms of the *Ciita* gene, which are regulated by different promoters (Fig. 7a)^33^. Indeed, freshly isolated SP ILC3s used *Ciita* promoters *pI*, *pIII*, and *pIV*, whereas ex vivo isolated SI ILC3s expressed only the *pI*-regulated isoform. These data indicate that there is a tissue-specific regulation of *Ciita* expression in ILC3s. IL-23 reduced the expression of promoter *pIII* in SP ILC3s (Supplementary Fig. 7a). It is hence possible that the IL-23-dependent repression of MHC II in vivo and in vitro was at least partially mediated by the reduction of *Ciita pIII*.

The promoter *pIV*, which was expressed in SP ILC3s, is inducible by IFN-γ^33^. IFN-γ expression was remarkably higher in the SP (Fig. 7b) pointing to its potential role in *Ciita pIV*-driven MHC II expression of SP ILC3s. To delineate the role of IFN-γ, we analyzed the expression of MHC II on SP and SI ILC3s of *Ifng*^*−/−*^ mice and after stimulation of SI ILC3s with IFN-γ. SP NCR^−^ ILC3s had a significantly lower expression of MHC II in *Ifng*^*−/−*^ mice as compared with WT mice (Fig. 7c and Supplementary Fig. 8). CD80 and CD86 expression levels were not affected (Supplementary Fig. 7b). Conversely, stimulation with IFN-γ significantly increased the expression of MHC II molecules and *Ciita* promoter *pIV* by SI NCR^−^ ILC3s (Fig. 7d, e). The effect on MHC II induction was abolished in cultures containing IFN-γ and IL-23 suggesting a dominant role of IL-23 in suppressing the expression of MHC II on ILC3s (Supplementary Fig. 7c). To analyse if IFN-γ also affects the capacity of ILC3s to stimulate T-cell responses, SI NCR^−^ ILC3s were incubated with IFN-γ or left untreated and used for stimulation of CD4^+^ T cells with Ova peptide (Fig. 7f). IFN-γ stimulated SI ILC3s were significantly better at inducing T-cell proliferation as compared with untreated cells.

In summary, we provide evidence that IFN-γ in the SP environment increases expression of MHC II molecules on NCR^−^ ILC3s and enhances their potential to induce CD4^+^ T-cell immune responses.

## Discussion

We show here that NCR^−^ ILC3s have a tissue-specific transcriptional signature. Transcripts associated with MHC II Ag presentation are relatively enriched in SP NCR^−^ ILC3s. SP NCR^−^ ILC3s are significantly more efficient in inducing CD4^+^ T-cell activation and proliferation as compared with SI NCR^−^ ILC3s. A subset of SI MHC II^+^ NCR^−^ ILC3s, however, can induce Ag-specific CD4^+^ T-cell proliferation. The fact that SI ILC3s can adopt an APC phenotype upon in vitro culture or in vivo migration to the SP suggests that environmental signals determine the capacity of ILC3s to present Ag and to stimulate CD4^+^ T cells. Indeed, we identified two essential pathways regulating ILC3-T-cell interactions in the SI vs. SP. One is mediated by microbe-induced IL-23, which supresses MHC II expression of NCR^−^ ILC3s through mTORC1 and STAT3 activation. The other pathway involves IFN-γ signaling, which induces the expression of MHC II on NCR^−^ ILC3s and promotes NCR^−^ ILC3-mediated CD4^+^ T-cell activation and proliferation. The IFN-γ induced MHC II expression of NCR^−^ ILC3s is reflected by the induction of *Ciita pIV* expression in ILC3s upon IFN-γ stimulation. The fact that the IFN-γ expression is low in the SI of WT mice explains why in *Ifng*^*−/−*^ mice no significant reduction of MHC II expression by SI ILC3s was observed. Likewise, IL-23 expression is low in the SP and deletion of *Il23p19* does not alter MHC II expression of SP ILC3s.

SI ILC3s were shown to suppress commensal bacteria-specific T-cell responses^12^. The discrepancy to our data might be partially explained by the fact that we studied ILC3 APC function under activation conditions. Similarly, a study published in this issue demonstrates that upon activation human ILC3s acquire Ag-presenting properties for the induction of Ag-specific CD4^+^ memory T-cell responses^34^. In mice upon IL-1β-stimulation both SI and SP ILC3s upregulate OX40L. OX40L has been described to be essential for the induction of pathogenic T-cell responses by TL1a-activated ILC3s in chronic colitis^35^. Our findings indicate that SI MHC II^+^ ILC3s do not block CD4^+^ T-cell immune responses per se. Under steady-state conditions, however, and under the local influence of microbiota and IL-23 the frequency of MHC II^+^APC-like ILC3s is probably too low to elicit T-cell responses in the SI. Moreover, our data strongly suggests a dominant effect of IL-23 in repressing MHC II expression of ILC3s.

During the early phase of viral infections, innate immune cells can rapidly release high amounts of IFN-γ. This could result in the expansion of SP MHC II^+^ ILC3s localized at the border between T and B-cell areas^36^, where Ag uptake and presentation takes place. Our findings that SI ILC3s upregulate MHC II and induce CD4^+^ T-cell proliferation after stimulation with IFN-γ demonstrate their potential to act as APCs under conditions where IFN-γ is released, e.g., during infections. Whether this is sufficient to promote CD4^+^ T-cell responses in the gut remains to be investigated.

It has been previously reported that the microbiota promotes IL-23-induced phosphorylation of STAT3, thereby inducing IL-22 in ILC3s^29^. We describe here that microbe-induced IL-23 also limits the frequency of MHC II^+^ NCR^−^ ILC3s in the SI. NCR^−^ ILC3s can be further subdivided into CCR6^+^ and CCR6^−^ cells. Interestingly, IL-23 has been shown to promote the development of T-bet^+^CCR6^−^NCR^−^ ILC3s^37^, which lack MHC II^12^. Our data strongly suggest that the IL-23-rich SI environment favors the accumulation of MHC II^−^ ILC3s, but amongst this subset we found both CCR6^+^ and CCR6^−^ ILC3s. IL-23 signaling in NCR^−^ ILC3s induced mTORC1 activation and STAT3 phosphorylation. We propose a model in which the collaborative mTORC1 and STAT3 signaling leads to downregulation of MHC II, most likely by the reduction of *Ciita* promoter *pIII* expression. In addition, IL-23 signaling may bypass mTORC1 to some extent and activate STAT3 via Janus kinases (JAK)^38^. We cannot exclude that other intestinal factors may prevent NCR^−^ ILC3-mediated CD4^+^ T-cell proliferation, in particular by inhibition of costimulatory or induction of coinhibitory molecules.

ILC3s are mainly tissue-resident cells and therefore exposed to local signals coming from other immune cell subsets, soluble factors, metabolites, and microbial products^39^. Recently, a tissue-specific imprinting has been reported for group 2 ILCs^18^ and group 1 ILCs^40^. Furthermore, Nussbaum et al. showed that the tissue environment affects the expression of several surface markers, e.g., NKp46 and NK1.1, by total ILC3s in SP and SI and that the SI environment inhibits the capacity of ILC3s to reject IL-12 secreting melanomas^17^. Our study provides a comprehensive comparison of transcriptomic profiles of NCR^−^ ILC3s uncovering a tissue-specific signature responsible for Ag presentation and T-cell stimulation. We show that the ratio of NCR^−^ ILC3 subsets with and without APC functions depends on IFN-γ and microbe-induced IL-23.

The discovery that APC properties of ILC3s are controlled by IFN-γ and microbe-induced IL-23 has important implications for our understanding of immune regulation in the gut. While the SP provides cytokines promoting the differentiation of MHC II^+^ ILC3s that are efficient inducers of T-cell responses, our data extend the current model in which intestinal T-cell responses are limited by ILC3s. We propose that under steady-state conditions microbial commensals trigger constitutive IL-23 production by mononuclear cells and subsequently reduction of MHC II^+^ ILC3s in the SI. Altogether this might be essential to promote tissue protection and to prevent T-cell-dependent immune responses. Under chronic inflammatory conditions, however, intestinal ILC3s may promote pathogenic pro-inflammatory T-cell activation as previously suggested^35^. Therefore, tissue-specific alterations of the cytokine repertoire and microbial composition during infectious or inflammatory diseases should be considered as critical factors regulating ILC3-T-cell responses and disease outcome.

## Methods

### Antibodies

Fluorescent-labeled or biotin-conjugated antibodies (Abs) were purchased from BioLegend, eBioscience, Cell Signaling Technology, LI-COR Biosciences or BD Bioscience. The following Abs were used anti-CD3ε (145-2C11), anti-CD4 (RM4-5 or GK1.5), anti-CD8α (53-6.7), anti-CD11b (M1/70), anti-CD11c (N418), anti-CD19 (6D5), anti-CD45R (RA3-6B2, B220), anti-CD69 (H1.2F3), anti-CD74 (CLIP), anti-CD80 (16-10A1), anti-CD86 (GL1), anti-CD90.2 (Thy1.2, 30-H12), anti-CD117 (2B8), anti-Gr-1 (RB6-8C5, Ly-6G), anti-TCR-β (H57-597), anti-TCR-γδ (UC7-13D5), anti-TER-119 (TER-119), anti-MHC II (M5/114.15.2), anti-NKp46 (29A1.4), anti-NK1.1 (PK136), anti-KLRG1 (2F1), anti-Ea_52-68_ (ebioYAe), anti-p-STAT3 (Tyr705) (13A3-1), anti-RORγt (AFKJS-9 or B2D), anti-OX40L (RM134L), anti-IFN-γ (XMG1.2), anti-TNF (MP6-XT22), anti-IL-22 (1H8PWSR), anti-tubulin (DM1A), anti-p-S6 (Ser235/236) (2F9), anti-p-S6 (Ser235/236) (D57.2.2E), anti-p-mTOR (Ser2448) (MRRBY), anti-mouse IgG IRDye680, and anti-rabbit IgG IRDye800.

### Mice

Breeding and maintenance of SPF mice were performed in the animal facilities of the Department of Biomedicine (University of Basel, Switzerland) and the Institute for Pharmacology and Toxicology (University of Veterinary Medicine Vienna, Austria; license: BMWFW-68.205/0093-WF/V/3b/2015). Germ-free (GF) mice and gnotobiotic sDMDMm2 mice were bred at the Clean Mouse Facility (University of Bern, Switzerland). GF mice were kept in flexible-film isolators or in individually ventilated cages (IVC). Routine monitoring by culture-dependent and -independent methods was done to confirm GF status. Gnotobiotic sDMDMm2 mice were generated by oral and rectal inoculation with a mixture of 12 bacterial strains (see Table 1)^26,41^. All animal experiments were conducted according to the Swiss Veterinary Law and Institutional Guidelines and were approved by the Cantonal Veterinary Office Basel City. Male or female mice of 8-13 weeks of age were used in all experiments. For all experiments, age- and sex-matched experimental groups of mice were used. Mice were housed under pathogen-free conditions in individually ventilated cages in a 22 °C temperature-controlled room with 12-h light–12-h dark cycles and free access to food and water. Experimental and control mice were co-housed and euthanized by terminal CO_2_ inhalation.

Conventional *C57BL/6* mice (WT) were purchased from Janvier Labs (Saint Berthevin Cedex, France). The following transgenic mouse strains on *C57BL/6* background were used: OT-II T-cell receptor transgenic *(OT-II*^*tg*^*)* mice (kindly provided by Antonius Rolink, University of Basel, Switzerland)^42^, *Rag2*^*−/−*^ mice (kindly provided by Georg Hollaender, University of Basel, Switzerland and Jesus College Oxford, UK)^43^, *Rag1*^*−/−*^ mice, *RORc(γt)-Cre*^*tg*^ mice (kindly provided by Andreas Diefenbach, Charité, Berlin, Germany)^44^, *Rosa26R*^*eYFP/+*^ (purchased from The Jackson Laboratory, JAX: 006148)^45^, *Il23p19*^*−/−*^ mice (kindly provided by B. Becher, University of Zurich, Switzerland)^46^, *Rag2*^*−/−*^*Il2rg*^*−/−*^ mice (kindly provided by Joerg Kirberg, Paul-Ehrlich Institut, Langen, Germany)^47^, *Flt3L*^*tg*^ mice (kindly provided by Antonius Rolink, University of Basel, Switzerland)^48^, *Myd88*^*fl/fl*^ mice (purchased from The Jackson Laboratory, JAX: 008888)^49^, and *Ifng*^*−/−*^ mice (kindly provided by Markus Heim, University of Basel, Switzerland)^50^. *CD45.1*^*+*^ mice (purchased from The Jackson Laboratory, JAX: 002014) were bred to *Rag2*^*−/−*^ mice.

To generate *Rorγt*^*fm+*^ mice, *RORc(γt)-Cre*^*tg*^ mice were bred to *Rosa26R*^*eYFP/+*^ mice, as published before^44^. In addition, *Rorγt*^*fm+*^ mice were bred to *Rag2*^*−/−*^ mice to generate *Rorγt*^*fm+*^*Rag2*^*−/−*^ mice. *RORc(γt)-Cre*^*tg*^ mice were bred to *Rptor*^*fl/fl*^ mice (kindly provided by Michael N. Hall, University of Basel, Switzerland)^51^ and backcrossed to *Rag2*^*−/−*^ background to generate *Rptor*^*ILC3−/−*^ mice. *VAV1-Cre*^*tg*^ mice^52^ were bred to *Stat3*^*fl/fl*^ mice^53^ to generate *VAV1-Cre*^tg^*Stat3*^*fl/fl*^ mice. *RORc(γt)-Cre*^*tg*^ mice were bred to *Myd88*^*fl/fl*^ mice to generate *Myd88*^*ILC3−/−*^ mice.

### Isolation of cells

SP and SI cells were isolated as published before^14^. In brief, the SI was opened longitudinally, incubated with 30 mM EDTA in 1 x PBS and washed several times with 1 x PBS to remove feces and mucus. Tissue pieces were digested in DMEM (Thermo Fisher Scientific) containing 0.025 mg per mL DNase I (Sigma-Aldrich) and 1 mg per mL collagenase D (Sigma-Aldrich) for 15 min at 37 °C. Supernatant was collected after washing with DMEM. Digestion steps were repeated three times with remaining tissue. After digestion SI cells were purified by Percoll density gradient (40/80%) centrifugation. The SP was cut into pieces and digested as the SI with DMEM containing DNase I and Collagenase D in 4 steps, each 15 min at 37 °C. Afterwards erythrolysis was performed.

Bone marrow-derived dendritic cells (BMDCs) were generated as published before^14^. In brief, femur and tibia were crushed. After erythrolysis bone marrow cells were cultured with Fms-related tyrosine kinase 3 ligand (Flt3lg)^54^ (kindly provided by Antonius Rolink, University of Basel, Switzerland) for 7 days at 37 °C, 10% CO_2_.

*OT-II*^*tg*^ CD4^+^ T cells were isolated from secondary lymphoid organs as described elsewhere^14^. In brief, suspensions of SP and lymph nodes were generated and erythrolysis was performed. CD4^+^ cells were enriched with CD4 Microbeads (Miltenyi Biotec) according to manufacturer’s instructions. To obtain a pure CD4^+^ T-cell population, CD11c^−^CD4^+^ T cells were sort-purified after enrichment. Sorted CD4^+^ T cells were stained with 7.5 µM Carboxyfluorescein succinimidyl ester (CFSE, Molecular Probes) at room temperature (10 min) and used for Ag presentation assays.

### Flow cytometry and cell sorting

Flow cytometry and cell sorting were done according to standard protocols. Single-cell suspensions were incubated with anti-FcγRII/RIII (cell culture supernatant of hybridoma cells producing clone 2.4G2^55^ (kindly provided by Hans Acha-Orbea, University of Lausanne, Switzerland) and surface marker-specific Ab for 30 min on ice in 1 x PBS containing 3% FBS. Live/dead staining was done with the fixable viability dyes eFluor 450 (eBioscience) or Zombie Aqua^TM^ (BioLegend). For intracellular RORγt staining cells were fixed and permeabilized with the FoxP3 transcription factor staining buffer set (eBioscience) according to manufacturer’s instructions. Cytokine staining was done after fixation with PBS containing 4% paraformaldehyde for 10 min on ice. For staining of p-STAT3 (Tyr705), p-mTOR (Ser2448) or p-S6 (Ser235/236) cells were fixed in PBS containing 4% paraformaldehyde for 15 min at 37 °C directly after stimulation. Permeabilization was done with True-Phos™ Perm Buffer (BioLegend) according to manufacturer’s instructions. Afterward staining was done for 30 min on ice.

Annexin V staining was done with Annexin V APC (BD Biosciences) according to manufacturer’s instructions.

To isolate NCR^−^ ILC3s by cell sorting, SP and SI cells were stained with Abs specific for CD117, Thy1.2, KLRG1 and a lineage cocktail (lin) of Abs. Unless stated otherwise the following Abs were used for the lin cocktail: CD3ε, CD8α, CD11b, CD11c, CD19, B220, Gr-1, TCR-β, TCR-γ/δ, TER-119, NK1.1, and NKp46. To isolate MHC II^+^ cells, additional staining with MHC II Ab was done.

Data were acquired with FACS Canto II (BD Bioscience), LSR Fortessa (BD Bioscience), or CytoFLEX (Beckman Coulter’s). Sorting was done with a FACS Aria II (BD Bioscience). Diva Software (BD FACS Aria II, BD LSR Fortessa and BD FACS Canto II) and CytExpert Software (CytoFLEX, Beckman Coulter) were used for data collection. For analysis FlowJo (Tree Star) software was used.

### Culture and stimulation of ILC3s

Cell culture was performed in IMDM medium (IMDM powder, Sigma-Aldrich), containing 3.02 g per L NaHCO_3_ (Sigma-Aldrich), 1% Penicillin Streptomycin (Thermo Fisher Scientific), 1% Ciproxine Perfusion 0.2 g (Bayer), 0.1% Kanamycin (Sigma-Aldrich), 1% Insulin Transferrin Selenium (Thermo Fisher Scientific), 0.3% Primatone (Sigma-Aldrich), 1% MEM-NEAA (Thermo Fisher Scientific), 0.1% 2-mercaptoethanol (Thermo Fisher Scientific), and 5% FBS. Sort-purified SI ILC3s (CD117^+^lin^−^Thy1.2^+^KLRG1^−^) were cultured at 37 °C, 10% CO_2_ for 7 days with 5% supernatant of IL-2 secreting cell line X63Ag8.653^56^ (kindly provided by Antonius Rolink, University of Basel, Switzerland), IL-7 (30 ng per mL, Preprotech), SCF (20 ng per mL, Preprotech), 0.5 µg per mL Amphotericin B (Sigma-Aldrich), and 2% penicillin streptomycin solution (Thermo Fisher Scientific) in 24-well plates (0.5–1 × 10^6^ cells per well). Medium was replaced by IMDM medium without Amphotericin B the next day. To analyse in vitro downregulation of MHC II and CD74 ILC3s were exposed to 20 ng per mL IL-23 (eBioscience) for 7 days at 37 °C, 10% CO_2_ in addition to IL-2, IL-7, and SCF. Fresh IL-23 was added every 2–3 days.

For analysis of *Ciita* promoter usage, MHC II expression and T-cell stimulation capacity NCR^−^ ILC3s (CD117^+^lin^−^Thy1.2^+^KLRG1^−^) were sort-purified from the SI and incubated in 24-well plates (0.5–1 × 10^6^ cells per well) with 20 ng per mL IFN-γ (eBioscience) for 48 h or 120 h without additional cytokines at 37 °C, 10% CO_2_. Combined stimulation with IFN-γ (20 ng per mL) and IL-23 (20 ng per mL) was done for 120 h without additional cytokines at 37 °C, 10% CO_2_.

For phospho-flow staining sort-purified NCR^−^ ILC3s (CD117^+^lin^−^Thy1.2^+^KLRG1^−^) were stimulated for the indicated time points with or without IL-23 (20 ng per mL) or IL-1β (20 ng per mL) in IMDM medium. To analyse *Ciita* promoter usage or induction of OX40L, ILC3s (CD117^+^lin^−^Thy1.2^+^KLRG1^−^) were isolated from *Rag2*^*−/−*^ mice and stimulated for 18 h with IL-23 (20 ng per mL) or IL-1β (20 ng per mL) in IMDM medium.

### Antigen presentation assay

ILC3s or in vitro generated BMDCs were stimulated overnight with 20 ng per mL IL-1β (Biovison Inc.). Unless otherwise indicated 5 × 10^4^ ILC3s or BMDCs were cultured with 1.5 × 10^5^ CFSE-labeled *OT-II*^*tg*^ CD4^+^ T cells in the presence of Ova protein (100 µg per mL, Imject Ovalbumin, Thermo Fisher Scientific, Inc.) or Ova_323-339_ peptide (5 µg per mL, AnaSpec) or medium alone for 72 h at 37 °C, 10% CO_2_. T cells were gated as CD3ε^+^ or CD117^−^; cells.

### Eα-GFP assay

TOP10 *E. coli* bacteria were transformed with a plasmid (kindly provided by Marc Jenkins, University of Minnesota, USA) encoding His-tagged Eα-GFP^57^. After expansion and lysis of bacteria, the protein was purified with a nickel column (His-Select Nickel Affinity Gel, Sigma-Aldrich) according to manufacturer’s instructions. Purity was controlled by polyacrylamid gel electrophoresis.

To analyse processing of Eα-GFP sort-purified NCR^−^ ILC3s (CD117^+^lin^−^Thy1.2^+^KLRG1^−^) from SP and SI were incubated with 100 µg per mL Eα-GFP protein in 96-well plates for 72 h at 37 °C, 10% CO_2_. Surface presentation of Eα_52-68_ peptide was analyzed by flow cytometry with the YAe antibody (ebioYAe).

### Adoptive cell transfer

Lin^−^Thy1.2^+^eYFP^+^ ILC3s of SP and SI were isolated from *RORγt*^*fm+*^*Rag2*^*−/−*^ mice. 0.5–1 × 10^6^ SI ILC3s and 1–2 × 10^5^ SP ILC3s were intravenously (i.v.) injected into *Rag2*^*−/−*^*Il2rg*^*−/−*^ mice. Five weeks after transfer SP and SI cells were isolated and analyzed by flow cytometry. For short-term migration assays (7 days), total splenocytes or total SI lymphocytes from *Rorγt*^*fm+*^*Rag2*^*−/−*^ mice were isolated following our protocols described before and i.v. injected into *Rag2*^*−/−*^*Il2rg*^*−/−*^ mice (1 × 10^7^ SI cells or 5 × 10^7^ SP cells per mouse). Lin^−^Thy1.2^+^KLRG1^−^eYFP^+^MHC II^+^ and Lin^−^Thy1.2^+^KLRG1^−^CD45.1^+^MHC II^−^ ILC3s were sort-purified from the SI of *Rorγt*^*fm+*^*Rag2*^*−/−*^ and CD45.1^+^*Rag2*^*−/−*^ mice, respectively. 1–2 × 10^5^ MHC II^+^ and MHC II^−^ ILC3s were i.v. injected into *Rag2*^*−/−*^*Il2rg*^*−/−*^ mice in a ratio of ~1:1. Five weeks after transfer SP and SI cells were isolated and analyzed by flow cytometry.

### RNA sequencing

Cell suspensions were generated from SP and SI of *RORγt*^*fm+*^ mice. For T- and B-cell depletion of splenocytes 1 × 10^7^ cells per mL were stained with CD3ε-biotin and B220-biotin Abs for 15 min on ice in PBS containing 3% FBS. Afterward, cells were incubated with Streptavidin Microbeads (Miltenyi Biotec) for 15 min on ice and depleted according to manufacturer’s instructions. CD117^+^lin^−^Thy1.2^+^eYFP^+^ NCR^−^ ILC3s were sort-purified from SI and SP suspensions. RNA was isolated from 1–2 × 10^4^ SP ILC3s and 0.5–3 × 10^5^ SI ILC3s per sample with the PicoPure RNA isolation kit (Thermo Fisher Scientific) according to manufacturer’s instructions. For each sample biological triplicates were generated.

*Rag2*^*−/−*^ mice were used for RNA sequencing of cultured ILC3s. CD117^+^Thy1.2^+^lin^−^(CD3ε, CD8α, CD11b, CD11c, CD19, B220, Gr-1, TCR-β, TCR-γ/δ, TER119, NK1.1)KLRG1^−^ ILC3s were sort-purified from SI and cultured for 7 days, as described above. RNA was isolated from cultured SI ILC3s (ca. 1 × 10^5^ ILC3s per sample) and freshly isolated CD117^+^lin^−^Thy1.2^+^KLRG1^−^ ILC3s of SP (0.2–0.5 × 10^5^ ILC3s per sample) and SI (ca. 1 × 10^5^ ILC3s per sample) with the PicoPure RNA isolation kit (Thermo Fisher Scientific). For each sample biological replicates were generated. RNA sequencing and quality control were done by the genomics facility of the Friedrich Miescher Institute for Biomedical Research (Basel, Switzerland) using Hiseq 2500 system. Total RNA was amplified with NuGEN Ovation RNA Seq System V2. Library preparation was done with Illumina TruSeq DNA Nano kit, according to manufacturer’s instructions. Base calling and quality scoring were performed using RTA software (Real-Time Analysis).

Obtained single-end RNA-seq reads were mapped to the mouse genome assembly, version mm9 (downloaded from genome.ucsc.edu), with RNA-STAR (2.5.0c)^58^, with default parameters except for reporting for multi-mappers only one hit in the final alignment files (outFilterMultimapNmax=1) and filtering reads without evidence in spliced junction table (outFilterType=“BySJout”). Subsequent gene expression data analysis was done within the R software (R Foundation for Statistical Computing, Vienna, Austria). Raw reads and mapping quality was assessed by the qQCreport function from the R/Bioconductor software package QuasR (version 1.14.0)^59^.

Using RefSeq mRNA coordinates from UCSC (genome.ucsc.edu, downloaded in July 2013) and the qCount function from QuasR package, we quantified gene expression as the number of reads that started within any annotated exon of a gene. The differentially expressed genes were identified using the edgeR package (version 3.16.5)^60^. Pools of mice (indicated as a factor) were included as a covariate. Genes with a false discovery rate (FDR) smaller than 0.05 and minimum log2 (fold change) of 1.5 were considered as differentially expressed.

### qRT PCR

For RNA isolation, tissue samples from duodenum, jejunum and terminal ileum of the SI and the whole SP were directly frozen in TRIzol (TRI Reagent, Thermo Fisher Scientific) in liquid N_2_. Homogenization of tissue was done in microtubes containing Zirconia beads (BioSpec Products) using FastPrep-24 instrument (MP Biomedicals) for 1 min at 6.5 m/s. RNA was isolated according to manufacturer’s instructions (TRi Reagent, Thermo Fisher Scientific). Quality and concentration were measured with a Nanodrop 2000c (Thermo Fisher Scientific). RNA of duodenum, jejunum, and ileum was pooled afterward, unless otherwise indicated.

Synthesis of the first-strand cDNA was done with Oligo dT (Promega), dNTPs (Roche), random hexamers (Sigma-Aldrich), and Superscript III Reverse Transcriptase (Invitrogen) according to manufacturer’s instructions. Quantitative real-time PCR (qRT PCR) was done using Sensimix SYBR-Hi-Rox Kit (Bioline) on a Rotor-Gene RG 3000A (Corbett Research). The following primer were used: *Tbp* fw (GGCACCACCCCCTTGTACCCT), *Tbp* rv (ACGCAGTTGTCCGTGGCTCT), *Il12b* fw (TGGGAGTACCCTGACTCCTG), *Il12b* rv (AGGAACGCACCTTTCTGGTT), *Tnfsf4* fw (ACTCTCTTCCTCTCCGGCAAA), *Tnfsf4* rv (TTGCCCATCCTCACATCTGG), *Il23p19* fw (CACCAGCGGGACATATGAATCT), *Il23p19* rv (CACTGGATACGGGGCACATT), *Ifng* fw (CTGAGACAATGAACGCTACAC), and *Ifng* rv (TTTCTTCCACATCTATGCCAC).

Gene expression was normalized to the gene encoding TATA box binding protein (Tbp) using the comparative threshold cycle method (ΔC_T_).

To analyse the expression of *pI*, *pIII*, or *pIV* regulated *Ciita* mRNA isoforms qRT PCR was done. SI and SP NCR^*−*^ ILC3s (Thy1.2^+^lin^−^KLRG1^−^) were isolated from *Rag2*^*−/−*^ mice. RNA was isolated with the RNeasy Micro Kit (Qiagen) according to manufacturer’s instructions. Quality control was done with a Nanodrop 2000c. cDNA synthesis and qRT PCR were done as described above. The following primer were used: *Ciita pI* fw (CAGGGACCATGGAGACCATAGT), *Ciita pI* rv (CAGGTAGCTGCCCTCTGGAG), *Ciita pIII* fw (CTGCATCACTCTGCTCTCTAA)*, Ciita pIII* rv (GTCATAGAGGTGGTAGAGATGT), *Ciita pIV* fw (CATGCAGGCAGCACTCAGAA), and *Ciita pIV* rv (GGGTCGGCATCACTGTTAAGG). Rotor-Gene software (Corbett Research) was used for data collection and analysis.

### In-Cell Western

Sort-purified lin^*−*^CD90.2^+^KLRG1^−^SI LC3s from *Flt3*^*tg*^ were cultured 48 h with or without 20 ng per mL IL-23 in the presence or absence of rapamycin (10 or 100 nM). After stimulation, the cells were fixed in PBS containing 3.75% paraformaldehyde for 30 min at room temperature and washed twice with 1% BSA in PBS. Then, cells were permeabilized for 30 min with 0.1% Triton X100 in 1% BSA/PBS and washed once. After blocking with 1% goat serum in 1% BSA/PBS, cells were incubated overnight with primary mouse α-tubulin (Sigma-Aldrich) and rabbit α-p-S6 Ser235/236 (Cell Signaling Technology) Abs. Finally, cells were washed three times with 0.1% Triton X100 in 1% BSA/PBS, incubated for 1 h with secondary goat α-mouse IgG IRDye680 (LI-COR Biosciences) and goat α-rabbit IgG IRDye800 (LI-COR Biosciences) Abs and washed five times with 0.1% Triton X100 in 1% BSA/PBS. Before scanning the cells with the Odyssey CLx Infrared Scanning System (LI-COR Biosciences), all supernatant was removed. Image Studio Software (LI-COR) using Odyssey CLx Infrared Scanning System (LI-COR Biosciences) was used for data collection.

### Statistical analysis

GraphPad Prism 7 (GraphPad Software, Inc., La Jolla, CA, USA) was used to calculate *P* values with one-way ANOVA, Mann–Whitney test or two-tailed Student’s *t* test with a 95% confidence interval, as outlined in each figure legend. In some experiments pools of mice were used for in vitro assays. Statistical analysis for these experiments was done with mixed-effects models (random effect: pool of mice, fixed effect: treatment, mouse strain or organ) and lmerTest package of R software with a Satterthwaite approximation^61^. For multiple comparisons with mixed effect models *P* values have been adjusted with Benjamini–Hochberg procedure.

### Reporting summary

Further information on research design is available in the Nature Research Reporting Summary linked to this article.

## Supplementary information

Supplementary Information

Reporting Summary

## Data Availability

Sequencing data reported in this paper is accessible with the following accession code: GSE114751 (https://www.ncbi.nlm.nih.gov/geo/query/acc.cgi?acc=GSE114751). Data bases used in this study are accessible at UCSC Genome Browser (http://hgdownload.soe.ucsc.edu/goldenPath/mm9/bigZips/, http://hgdownload.soe.ucsc.edu/goldenPath/mm9/database/). The data underlying Figs. 1e, 2a–d, 3a, b, 4c–e, 5a–g, 6a–d, 7a–f and Supplementary Figs. 2c, d, g, h, 3a, b, e, 4a, b, 5a, g, 6b, c, 7a–c and 8a, b are provided as a Source Data File.

## Source Data

Source Data

## Code availability

Data analyses codes have been published before and are available from the corresponding author on request^58–60^.
