# Supplementary material for: Microbiota-induced tissue signals regulate ILC3-mediated antigen presentation
Source: Nat Commun. 2020 Apr 14;11:1794. doi: 10.1038/s41467-020-15612-2 (PMC7156681; doi:10.1038/s41467-020-15612-2)
Supplement: Supplementary file 1 — Supplementary Information [file 41467_2020_15612_MOESM1_ESM.pdf]

Supplementary information for:
Microbiota-induced tissue signals regulate ILC3-mediated antigen presentation

Lehmann et al.

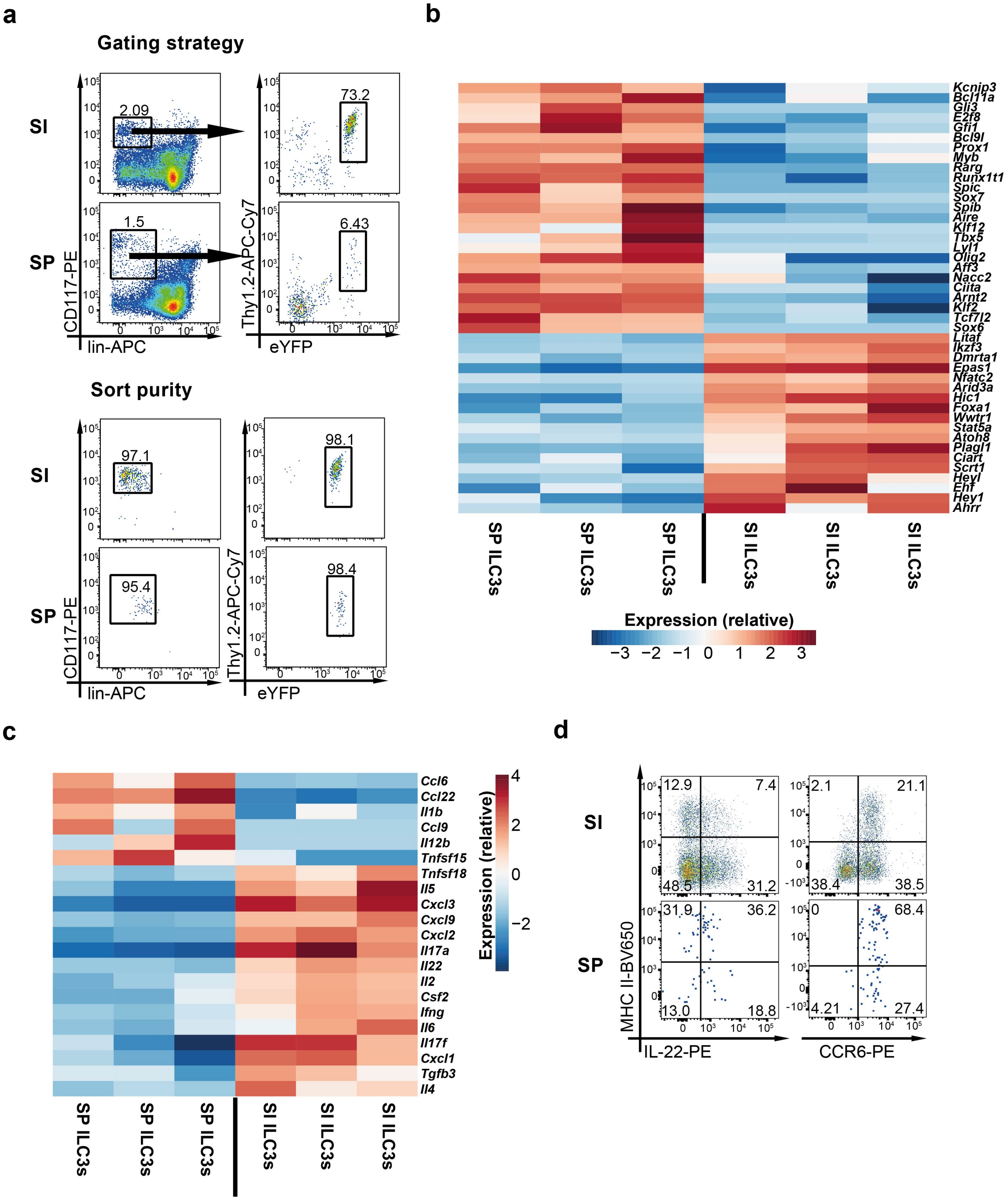

Supplementary Figure 1. Phenotype of splenic and intestinal ILC3s

(a) Gating strategy and sort purity of SI and SP ILC3s with regards to the RNA sequencing experiment. Lineage: CD3ε, CD8α, CD19, CD11b, CD11c, B220, Gr-1, TCR-β, TCR-γ/δ, TER119, NK1.1, NKp46. Corresponds to Figure 1, Figure 3 and Supplementary Figure 3. (b) Genes encoding transcription factors and (c) cytokines significantly differentially expressed in SP

and SI NCR$^-$ ILC3s (FDR<0.05, log2(fold change)>2) (d) SI and SP cells were isolated from *WT* mice. Naïve NCR$^-$ILC3s (gating as shown in Figure 1e) were analysed for expression of IL-22 and CCR6. Representative examples of 7 (IL-22) and 6 mice (CCR6) analysed in 2 independent experiments. Source data are provided as a Source Data File.

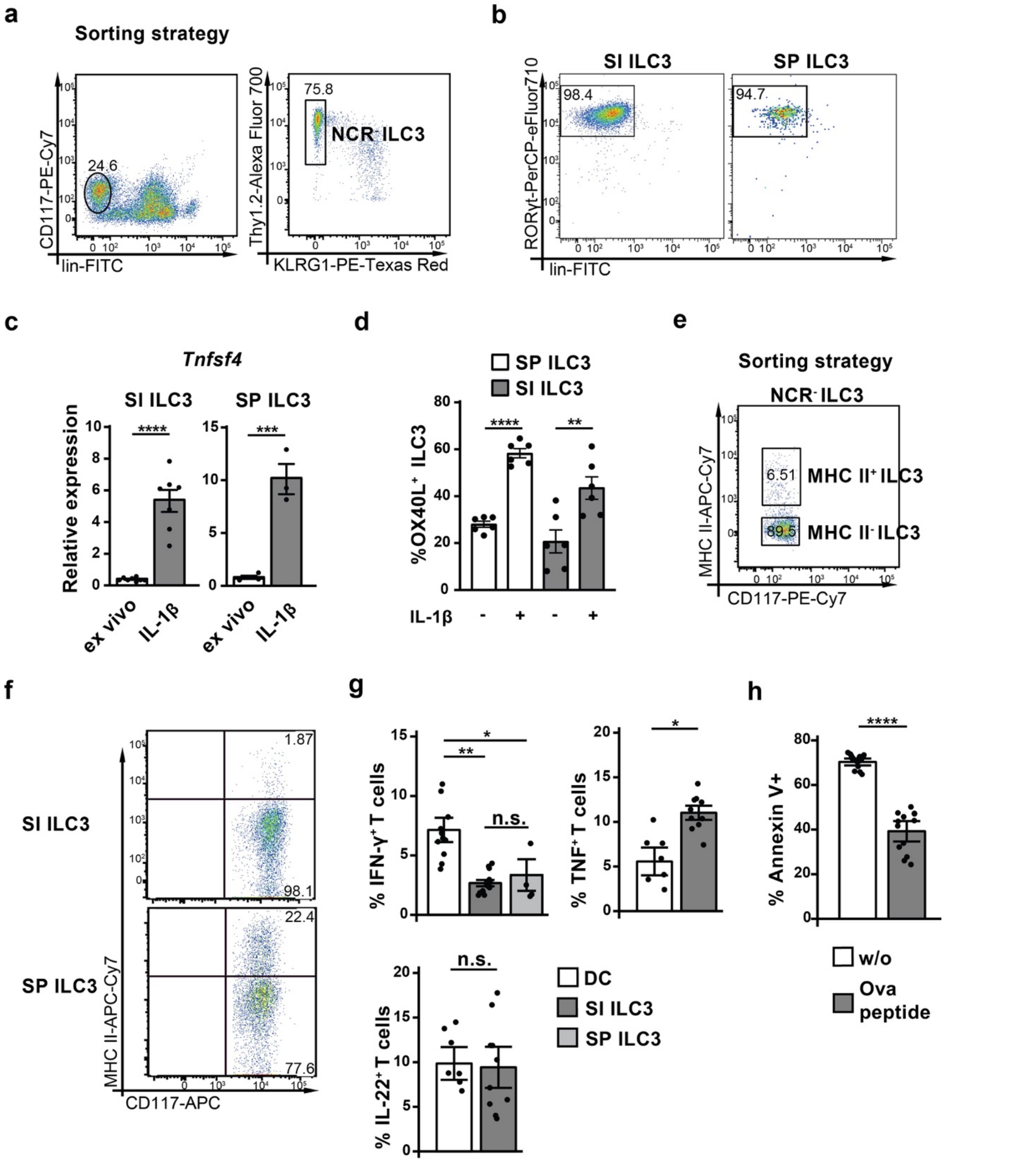

Supplementary Figure 2. Sorting strategy and T cell stimulation capacity of ILC3s

(a) Sorting strategy for NCR- ILC3s (depicted for *Rag2$^{-/-}$* SI ILC3s). Corresponds to Figures 2, 4-7, Supplementary Figures 2 and 5-7. (b) RORγt expression of ILC3s (*Rag2$^{-/-}$*). Representative example of 3 independent experiments. (c) Expression of *Tnfsf4* analysed by qRT PCR from sorted SI and SP ILC3s (*Rag2$^{-/-}$*) directly *ex vivo* (n=8 distinct SI, 4 distinct SP ILC3 samples) or after stimulation with IL-1β for 18 h (n=7 distinct SI, 3 distinct SP ILC3 samples). 5 (SI ILC3) and

3 (SP ILC3) independent experiments. (d) Expression of OX40L by Thy1.2^{+}lin^{-} CD117^{+}RORγt^{+}MHCII^{+} SI and SP ILC3s (*Rag2*$^{-/-}$) stimulated with or without IL-1β for 18 h. n=6 distinct samples of 3 independent experiments. (e) Sorting strategy for MHC II^{+} or MHC II^{-} NCR^{-} ILC3s. NCR^{-} ILC3s were gated as shown in Supplementary Figure 2a. Corresponds to Figure 2D and Supplementary Figures 3c, e. (f) MHC II expression of ILC3s sort-purified as MHC II^{-} after IL-1β activation and culture with *OT-II*^{tg} CD4^{+} T cells. ILCs were gated as CD117^{+}CFSE^{-}. Representative example of 4 individual experiments. (g) *OT-II*^{tg} CD4^{+} T cells were cultured either with 5 x 10^{4} IL-1β-activated SP or SI ILC3s (*Rag2*$^{-/-}$) or BMDCs in the presence of Ova peptide for 72h. Expression of IFN-γ (n=13 (DC), n=15 (SI ILC3), n=4 (SP ILC3) distinct samples), TNF (n=7 (DC), n=10 (SI ILC3) distinct samples) and IL-22 (n=7 (DC), n=10 (SI ILC3) distinct samples) by CD3^{+}CD117^{-}CD4^{+}Thy1.2^{+}CFSE^{+} T cells. 3-4 independent experiments. (h) *OT-II*^{tg} CD4^{+} T cells were cultured with IL-1β-activated SI ILC3s (*Rag2*$^{-/-}$) with or without (w/o) Ova peptide for 72h. Annexin V staining of T cells gated as CD3^{+} or CD117^{-}. n=16 (w/o) and n=13 (peptide) distinct samples. 3 independent experiments. Each symbol represents a sample and the bar graph represents the mean ± s.e.m.. n.s. not significant; **P≤0.05; **P≤0.01*; ****P≤0.001*; *****P≤0.0001*, calculated with two-tailed unpaired Student's t test (c), one-way ANOVA (two-tailed) and Bonferroni's multiple comparisons test (d) or mixed-effects models (two-sided) using lmerTest (g, h). Source data are provided as a Source Data File.

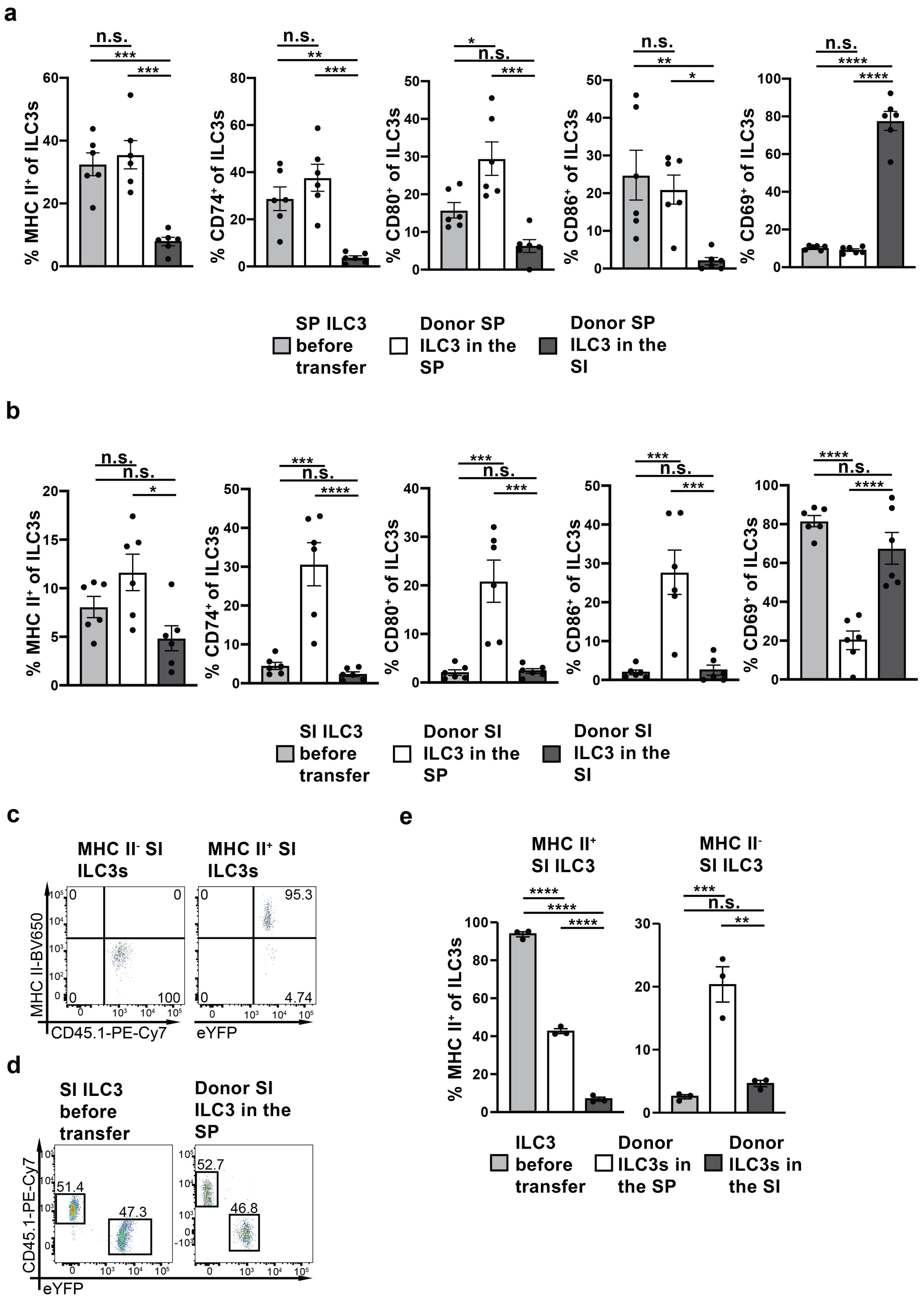

Supplementary Figure 3. Tissue-dependent regulation of ILC3 properties

Total splenocytes (a) or total SI lymphocytes (b) from *Rorγt*$^{fm+}$*Rag2*$^{-/-}$ mice were i.v. injected into *Rag2*$^{-/-}$*Il2rg*$^{-/-}$ mice. Cells were sort-purified as depicted in Supplementary Figure 1a. Expression of MHC II, CD80, CD86, CD74 and CD69 was analysed before transfer and 7 days after transfer on donor derived cells. ILC3s were gated as depicted in Figure 3a. n=6 distinct samples of 3 independent experiments. (c, d, e) MHC II$^-$ and MHC II$^+$ SI ILC3s were isolated from CD45.1$^+$ *WT* mice and *Rorγt*$^{fm+}$*Rag2*$^{-/-}$ mice respectively as depicted in Supplementary Figure 1a and 2e. (c) Sort purity of SI MHC II$^+$ (eYFP$^+$) and SI MHC II$^-$ (CD45.1$^+$) ILC3s. Representative example of 3 independent experiments. (d) SI MHC II$^+$ (eYFP$^+$) and SI MHC II$^-$ (CD45.1$^+$) ILC3s were i.v. injected into *Rag2*$^{-/-}$*Il2rg*$^{-/-}$ mice in a defined ratio. 5 weeks after transfer splenocytes were analysed by flow cytometry for CD45.1 and eYFP expression. Cells were gated as Thy1.2$^+$. Representative dot plots of 3 independent experiments. (e) MHC II$^-$ and MHC II$^+$ ILC3s were i.v. injected into *Rag2*$^{-/-}$*Il2rg*$^{-/-}$ mice. 5 weeks after transfer the MHC II expression of donor-derived ILC3s in the SP and SI of recipient mice was analysed. ILC3s were gated as Thy1.2$^+$lin$^-$eYFP$^+$ (sorted as MHC II$^+$) or Thy1.2$^+$lin$^-$CD45.1$^+$ (sorted as MHC II$^-$). n=3 distinct samples of 3 independent experiments. Each symbol represents a sample and the bar graph represents the mean ± s.e.m.. n.s. not significant; *$P \leq 0.05$; **$P \leq 0.01$; ***$P \leq 0.001$; ****$P \leq 0.0001$, calculated with one-way ANOVA (two-tailed) and Bonferroni's multiple comparisons test (a, b and e). Source data are provided as a Source Data File.

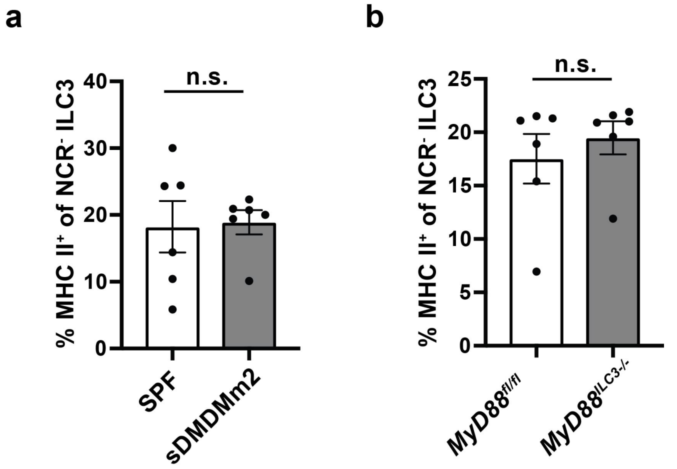

Supplementary Figure 4. Downregulation of MHC II depends on sDMDMm2 procaryotes but not on MyD88-mediated TLR signaling

(a-b) MHC II expression of SI NCR$^-$ ILC3s from *WT* GF mice colonized with sDMDMm2 bacterial strains or control SPF *WT* mice (a) and of SI NCR$^-$ ILC3s from *Myd88*$^{fl/fl}$ or *Myd88*$^{ILC3-/-}$ mice (b). n=6 mice analysed in 2 independent experiments. NCR$^-$ ILC3s were gated as shown in Figure 1. Each symbol represents a sample and the bar graph represents the mean ± s.e.m.. n.s. not significant, calculated with two-tailed Mann-Whitney test (a) or two-tailed unpaired Student's t test (b). Source data are provided as a Source Data File.

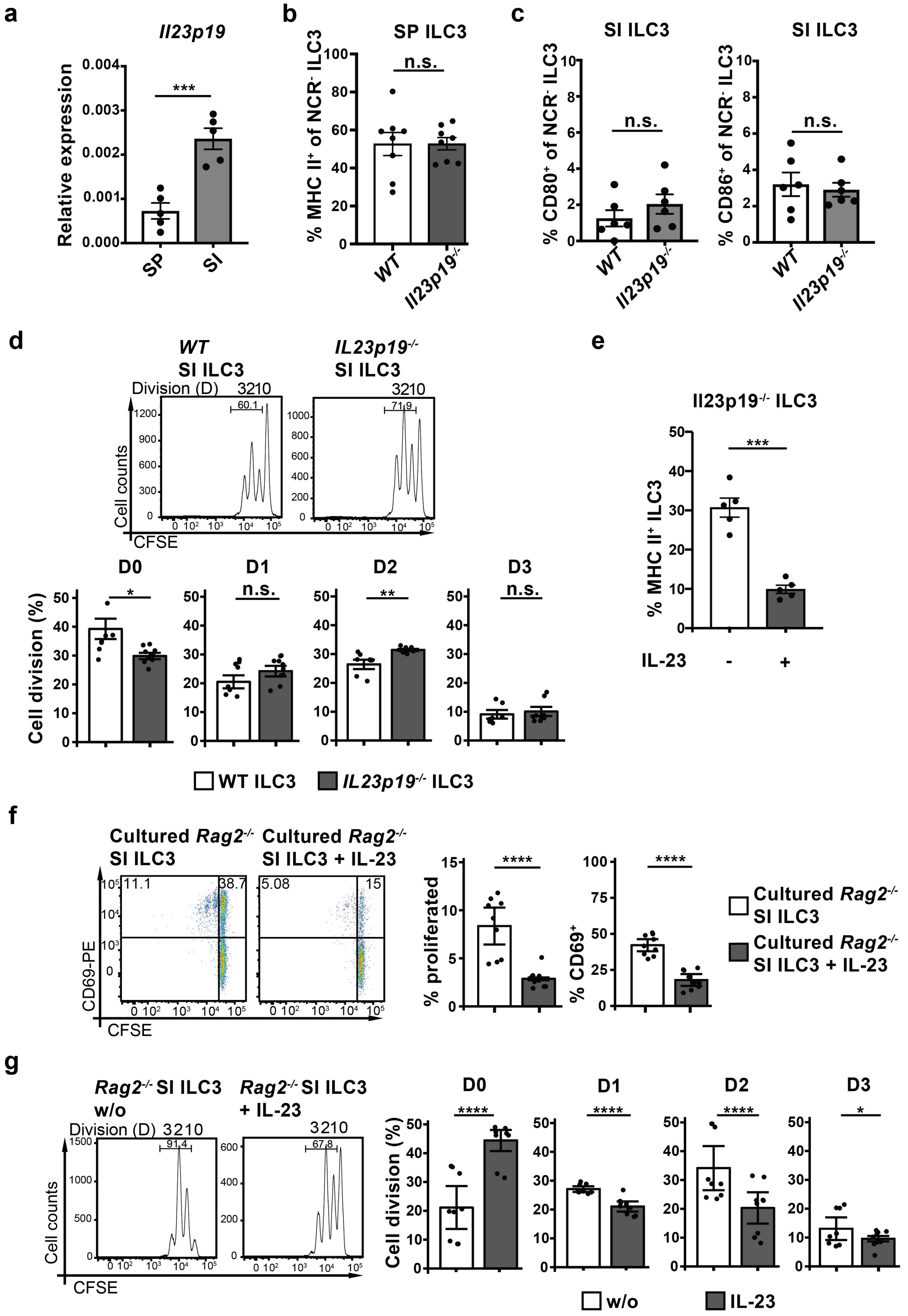

Supplementary Figure 5. Effects of IL-23 on ILC3s

(a) Whole organ RNA of SP and SI from *WT* mice was analysed by qRT PCR for expression of *Il23p19.* n=5 mice analysed in 2 independent experiments. (b) MHC II expression of SP ILC3s (gating as shown in Figure 1) from *WT* and *Il23p19*$^{-/-}$ mice (n=8 mice). 4 independent experiments. (c) CD80 and CD86 expression of *WT* and *Il23p19*$^{-/-}$ SI ILC3s (gating as shown in Figure 1). n=6 mice analysed in 2 independent experiments. (d) Naïve CFSE-labeled *OT-II*^{tg} $CD4^+$ T cells were cultured either with 5 x 10^4 SI ILC3s (sorted as depicted in Supplementary Figure 2a) from *WT* (n=8 distinct samples) or *Il23p19*$^{-/-}$ (n=10 distinct samples) mice in the presence of Ova peptide. Percentage of T cells which underwent 0, 1, 2 or 3 divisions (D0-D3). T cells were gated as $CD3^+$ or $CD117^-$. 4 independent experiments. (e) MHC II expression of *Il23p19*$^{-/-}$ ILC3s (sorted as shown in Supplementary Figure 2a) cultured 7 days with or without IL-23 (n=5 distinct samples). 3 independent experiments. (f, g) SI ILC3s were sort-purified (Supplementary Figure 2a) from *Rag2*$^{-/-}$ mice and cultured 7 days with or without IL-23 in addition to IL-2, IL-7 and SCF. (f) T cell proliferation and CD69 expression of CFSE-labeled *OT-II*^{tg} $CD4^+$ T cells stimulated with 5 x 10^4 cultured *Rag2*$^{-/-}$ SI ILC3s (sorted as depicted in Supplementary Figure 2a) in the presence of Ova protein. T cells were gated as $CD3^+$ or $CD117^-$. (g) Percentage of T cells which underwent 0, 1, 2 or 3 divisions (D0-D3) in co-culture with SI ILC3s (sorted as depicted in Supplementary Figure 2a) in the presence of Ova peptide. T cells were gated as $CD3^+$ or $CD117^-$. n=9 distinct samples of 3 independent experiments. Each symbol represents a sample and the bar graph represents the mean ± s.e.m.. n.s. not significant; *P≤0.05; ***P≤0.01*; ****P≤0.001*; *****P≤0.0001,* calculated with two-tailed unpaired (a, b, c) or two-tailed paired (e) Student's t test or mixed-effects models (two-sided) using lmerTest (d, f and g). Source data are provided as a Source Data File.

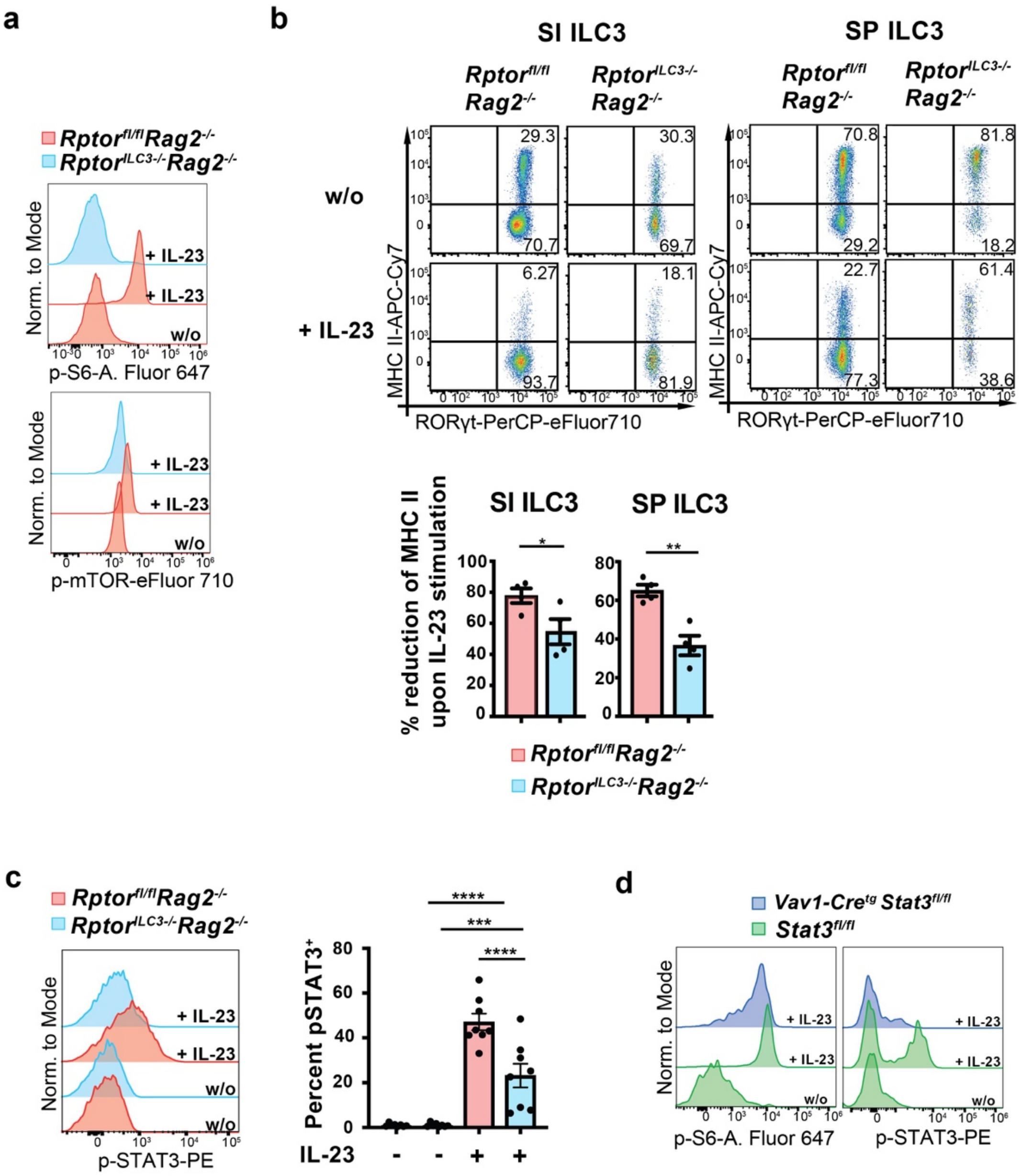

Supplementary Figure 6. mTORC1 and STAT3 mediate downregulation of MHC II in ILC3s

(a) Phosphorylation of S6 (Ser235/236) and mTOR (Ser2448) of sort-purified SI NCR$^-$ ILC3s (sorting strategy as shown in Supplementary Figure 2a) from *Rptor$^{fl/fl}$Rag2$^{-/-}$* (red) and *Rptor$^{ILC3-/-}$ Rag2$^{-/-}$* (blue) mice. ILC3s were stimulated for 17 h with IL-23 or were left untreated. Representative histograms from 4 independent experiments. Cells were gated as Thy1.2$^+$. (b) MHC II expression of ILC3s from *Rptor$^{fl/fl}$Rag2$^{-/-}$* (red) and *Rptor$^{ILC3-/-}$Rag2$^{-/-}$* (blue) mice cultured 7 days with or without IL-23. NCR$^-$ ILC3s were gated as shown in Figure 1. n=4 distinct samples of 4 independent experiments. The lower panel shows the relative reduction of MHC II upon IL-23 stimulation. (c) Phosphorylation of STAT3(Tyr705) of sort-purified (as depicted in

Supplementary Figure 2a), IL-23-stimulated or unstimulated SI ILC3s from *Rptor*$^{fl/fl}$*Rag2*$^{-/-}$ and *RptorILC3*$^{-/-}$*Rag2*$^{-/-}$ mice. ILC3s were stimulated for 20 minutes and gated as Thy1.2^{+}NKp46^{-}. n=8 distinct samples of 4 independent experiments. (d) Phosphorylation of STAT3 (Tyr705) and S6 (Ser235/236) of sort-purified (as depicted in Supplementary Figure 2a) SI NCR^{-} ILC3s from *Stat3*$^{fl/fl}$ (green) and *Vav1-Cre*^{tg}*Stat3*$^{fl/fl}$ (blue) mice. ILC3s were stimulated for 17 h with IL-23 or were left untreated. Representative histograms of 7 mice per group analysed in 2 independent experiments. Cells were gated as Thy1.2^{+}. Each symbol represents a sample and the bar graph represents the mean ± s.e.m.. **P≤0.05*; ***P≤0.01; ***P≤0.001*; *****P≤0.0001*, calculated with two-tailed unpaired Student's t test (b) or with one-way ANOVA (two-tailed) and Bonferroni's multiple comparisons test (c). Source data are provided as a Source Data File.

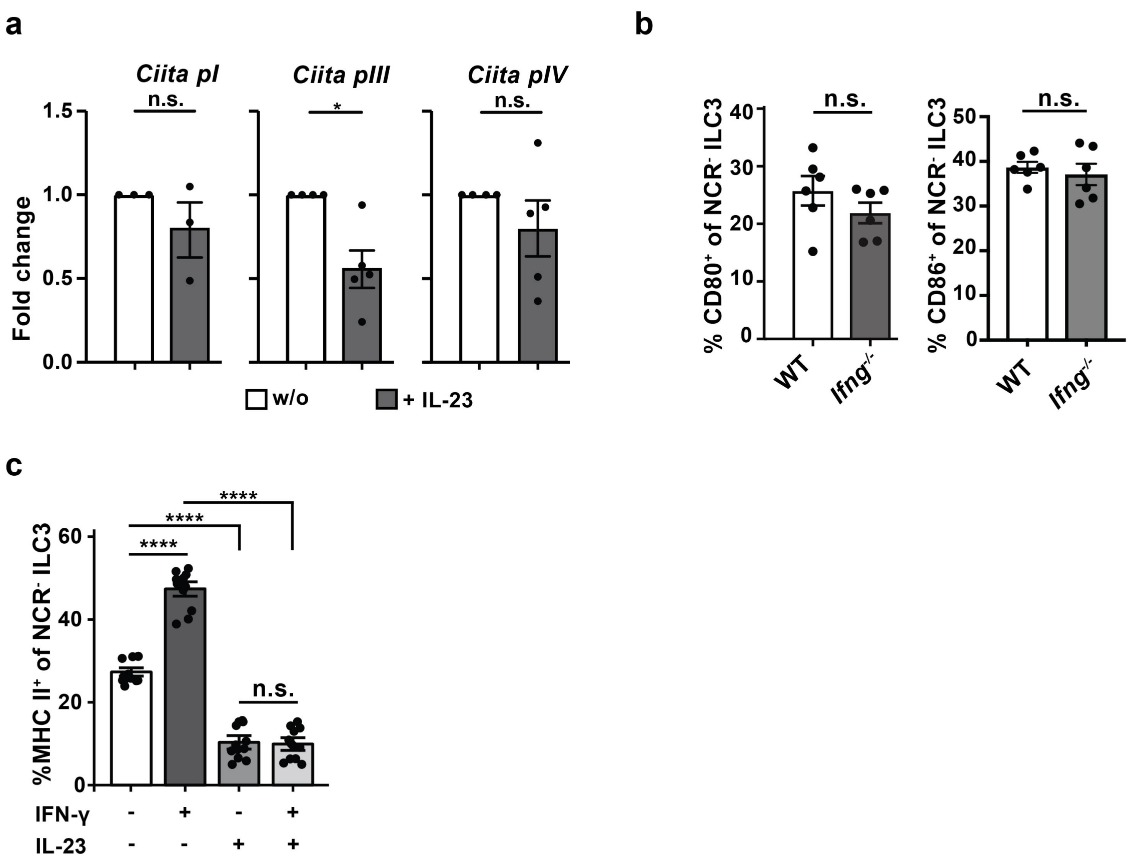

Supplementary Figure 7. Effect of IL-23 on *Ciita* expression and of IFN-γ on costimulatory molecules

(a) Fold change of the expression of *Ciita* mRNA isoforms regulated by promoter *pI* (n=3 distinct samples), *pIII* (n=4 (w/o) and n=5 (IL-23) distinct samples) and *pIV* (n=4 (w/o) and n=5 (IL-23) distinct samples) in isolated SP ILC3s (as depicted in Supplementary Figure 2a) stimulated 18 h with or without (w/o) IL-23. 3-4 independent experiments. (b) CD80 and CD86 expression of SP ILC3s (gating as shown in Figure 1) isolated from *WT* and *Ifng*$^{-/-}$ mice (n=6 mice analysed in 2 independent experiments). (c) MHC II expression of SI ILC3s (isolated from *Rag2*$^{-/-}$ as depicted in Supplementary Figure 2a) stimulated for 120 h (n=12 distinct samples of 3 independent experiments) with or without (w/o) IFN-γ and IL-23 as indicated in the figure. Each symbol represents a sample and the bar graph represents the mean ± s.e.m, n.s. not significant; *$P \leq 0.05$; ****$p \leq 0.0001$*, calculated with Mann-Whitney test (a), two-tailed unpaired Student's t test (b) or mixed-effects models (two-sided) using lmerTest (c). Source data are provided as a Source Data File.

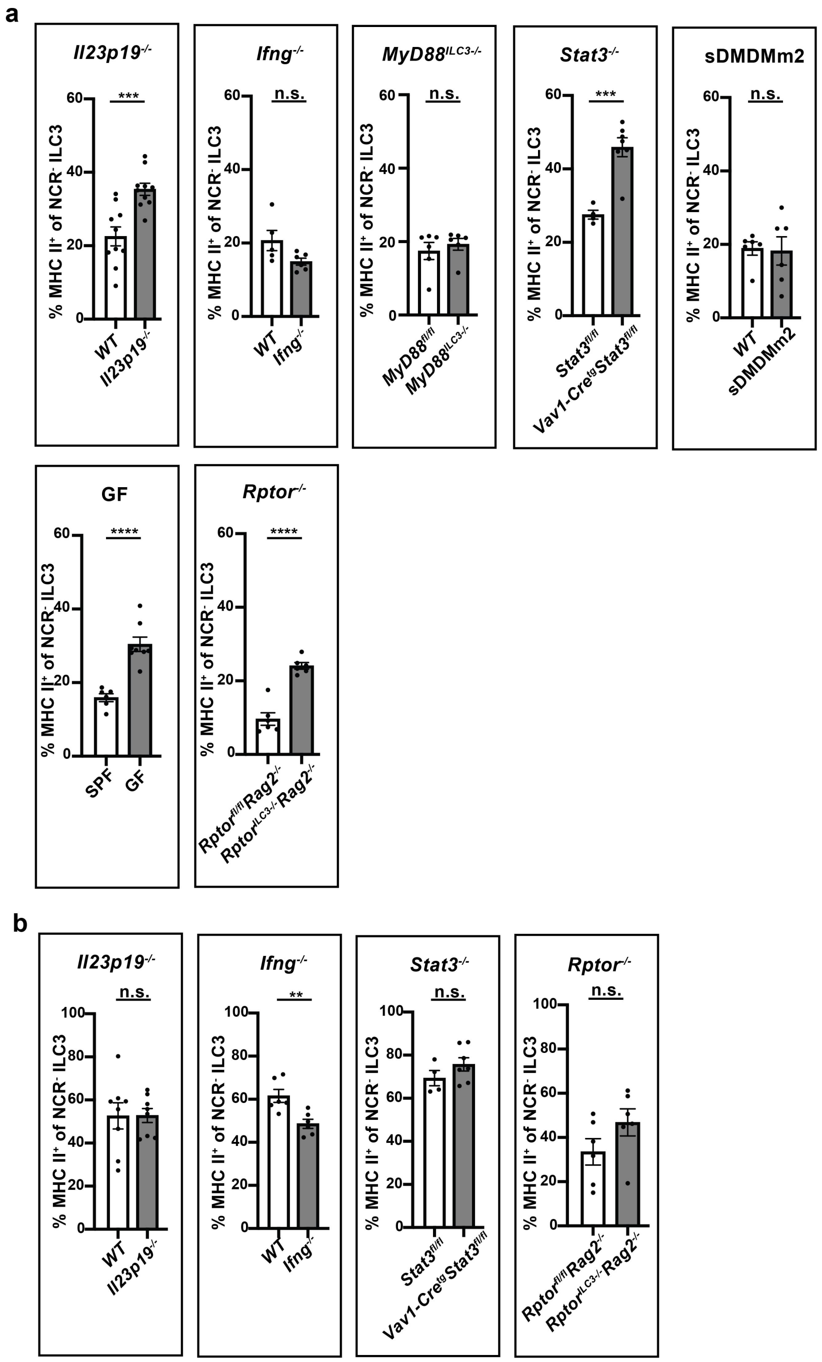

Supplementary Figure 8. Summary of MHC II expression of ILC3s of all mouse strains

Summary figure of MHC II expression of SI ILC3s (a) and SP ILC3s (b) from various mouse strains on *WT* and *Rag*$^{-/-}$ background. ILC3s were gated as shown in Figure 1e. See also Figures 5b, 5d, 6c, 6d, 7c, Supplementary Figures 4a, 4b and 5b for experimental details. Each symbol represents a sample and the bar graph represents the mean ± s.e.m.. n.s. not significant; ***P≤0.01; ***P≤0.001*; *****P≤0.0001*, calculated with two-tailed unpaired Student's t

test (*Il23p19*$^{-/-}$, *Ifng*$^{-/-}$, *Myd88*$^{ILC3-/-}$, *Stat3*$^{-/-}$, GF, *Rptor*$^{-/-}$) or two-tailed Mann-Whitney test (sDMDMm2). Source data are provided as a Source Data File.
